# Aorta in Pathologies May Function as an Immune Organ by Upregulating Secretomes for Immune and Vascular Cell Activation, Differentiation and Trans-Differentiation—Early Secretomes may Serve as Drivers for Trained Immunity

**DOI:** 10.3389/fimmu.2022.858256

**Published:** 2022-03-07

**Authors:** Yifan Lu, Yu Sun, Keman Xu, Fatma Saaoud, Ying Shao, Charles Drummer, Sheng Wu, Wenhui Hu, Jun Yu, Satya P. Kunapuli, John R. Bethea, Roberto I. Vazquez-Padron, Jianxin Sun, Xiaohua Jiang, Hong Wang, Xiaofeng Yang

**Affiliations:** ^1^ Cardiovascular Research Center, Departments of Cardiovascular Sciences and Biomedical Education and Data Sciences, Temple University Lewis Katz School of Medicine, Philadelphia, PA, United States; ^2^ Center for Metabolic Disease Research, Departments of Cardiovascular Sciences and Biomedical Education and Data Sciences, Temple University Lewis Katz School of Medicine, Philadelphia, PA, United States; ^3^ Sol Sherry Thrombosis Research, Departments of Cardiovascular Sciences and Biomedical Education and Data Sciences, Temple University Lewis Katz School of Medicine, Philadelphia, PA, United States; ^4^ Department of Biology, College of Arts and Sciences, Drexel University, Philadelphia, PA, United States; ^5^ DeWitt Daughtry Family Department of Surgery, Leonard M. Miller School of Medicine, University of Miami, Miami, FL, United States; ^6^ Department of Medicine, Center for Translational Medicine, Thomas Jefferson University, Philadelphia, PA, United States

**Keywords:** endothelial cell, canonical and noncanonical secretomes, inflammation, coronavirus infection, DAMPs

## Abstract

To determine whether aorta becomes immune organ in pathologies, we performed transcriptomic analyses of six types of secretomic genes (SGs) in aorta and vascular cells and made the following findings: 1) 53.7% out of 21,306 human protein genes are classified into six secretomes, namely, canonical, caspase 1, caspase 4, exosome, Weibel–Palade body, and autophagy; 2) Atherosclerosis (AS), chronic kidney disease (CKD) and abdominal aortic aneurysm (AAA) modulate six secretomes in aortas; and Middle East Respiratory Syndrome Coronavirus (MERS-CoV, COVID-19 homologous) infected endothelial cells (ECs) and angiotensin-II (Ang-II) treated vascular smooth muscle cells (VSMCs) modulate six secretomes; 3) AS aortas upregulate T and B cell immune SGs; CKD aortas upregulate SGs for cardiac hypertrophy, and hepatic fibrosis; and AAA aorta upregulate SGs for neuromuscular signaling and protein catabolism; 4) Ang-II induced AAA, canonical, caspase 4, and exosome SGs have two expression peaks of high (day 7)-low (day 14)-high (day 28) patterns; 5) Elastase induced AAA aortas have more inflammatory/immune pathways than that of Ang-II induced AAA aortas; 6) Most disease-upregulated cytokines in aorta may be secreted *via* canonical and exosome secretomes; 7) Canonical and caspase 1 SGs play roles at early MERS-CoV infected ECs whereas caspase 4 and exosome SGs play roles in late/chronic phases; and the early upregulated canonical and caspase 1 SGs may function as drivers for trained immunity (innate immune memory); 8) Venous ECs from arteriovenous fistula (AVF) upregulate SGs in five secretomes; and 9) Increased some of 101 trained immunity genes and decreased trained tolerance regulator IRG1 participate in upregulations of SGs in atherosclerotic, Ang-II induced AAA and CKD aortas, and MERS-CoV infected ECs, but less in SGs upregulated in AVF ECs. IL-1 family cytokines, HIF1α, SET7 and mTOR, ROS regulators NRF2 and NOX2 partially regulate trained immunity genes; and NRF2 plays roles in downregulating SGs more than that of NOX2 in upregulating SGs. These results provide novel insights on the roles of aorta as immune organ in upregulating secretomes and driving immune and vascular cell differentiations in COVID-19, cardiovascular diseases, inflammations, transplantations, autoimmune diseases and cancers.

## Introduction

Atherosclerosis is a dominant and increasing cause of mortality and morbidity worldwide. As a significant molecular mechanism, inflammation is a pathology from inception to the emergency of complications (1–3), which has been validated in atherogenesis by the findings from the Canakinumab Anti-inflammatory Thrombosis Outcomes Study (CANTOS): inhibition of interleukin-1β (IL-1β) reduces atherosclerotic cardiovascular disease (4, 5). Aortic arch atherosclerosis as a major atherosclerotic site (6) is an important source of embolic stroke (7). We reported that hyperlipidemia drives early atherosclerosis *via* disease risk sensing caspase 1-inflammasome pathway (8–12). In addition, abdominal aortic aneurysms (AAA), occurred in up to 8% of men aged >65 years, remain asymptomatic until they rupture. Ruptured AAA and its associated catastrophic pathological insult carry overall mortality in excess of 80% and 2% of all deaths are AAA-related. Pathologically, AAA is associated with inflammation, smooth muscle cell apoptosis, and matrix degradation (13). During AAA progression, inflammatory vascular smooth muscle cell (VSMC) populations, inflammatory monocytes and macrophages are significantly increased in aortic lesions (14). Moreover, chronic kidney disease (CKD) is associated with accelerated cardiovascular disease (CVD) even when kidney function is only mildly impaired; and the relative risk of all-cause death is increased by approximately threefold in individuals with a serum creatinine level greater than 150 μmol/l (15). Our reports showed that CKD increases vascular inflammation *via* uremic toxins as danger associated molecular patterns (DAMPs) (16–19)-caspase 1-inflamamsome (20) mediated pathway; and secretomes in peripheral blood mononuclear cells (PBMC) may play significant roles in promoting inflammation in CKD transition to end stage renal disease (21). Recently, we also reported that CD4^+^Foxp3^+^ regulatory T cells (Treg) (22–25) may use various secretomes to maintain Treg-ness (Treg suppression) (25), modulate anti-tumor immune responses (26), facilitate tissue repair (26) and stem cell-like functions in tissue regeneration (27). However, significant questions remain whether aorta serves as an immune organ by upregulating secretomes during atherosclerosis, AAA, and CKD; and whether aortic cells use different secretomes to fulfill pathological functions in different time courses.

Due to this vast coverage and the nature of being the first cell type to encounter any pathogen associated molecular patterns (PAMPs)/DAMPs in the blood circulation, the endothelium (endothelial cells, ECs) (28) could function as the primary intravascular sentinel system (9, 29, 30), which is similar to mucous membranes and skin to prevent microbial infections originally defined for immune system (https://clinicalinfo.hiv.gov/en/glossary/immune-system). Thus, we compared ECs to prototypic innate immune cells such as macrophages (31) in more than 10 innate immune features, and proposed a new paradigm that ECs are innate immune cells (2, 3, 32), which: 1) serve as sentinels of the innate immune system; 2) acquire function as antigen-presenting cells; 3) play immune enhancing and immune suppressive roles depending on their cytokine secreting panel; and 4) have plasticity to switch into other cell types (3, 32). In support to our model, we recently characterized transcriptomic changes of 1,311 innate immune regulatomic genes (33, 34) in ECs infected with coronaviruses, and stimulated by DAMPs in 28 EC microarray datasets with 7 monocyte datasets as controls. The expressions of innate immune regulators (IGs) are modulated in 21 human EC transcriptomic datasets by various PAMPs/DAMPs, namely, Middle East Respiratory Syndrome Coronavirus [MERS-CoV, a virus homologous to Severe Acute Respiratory Syndrome Coronavirus-2 (SARS-CoV2, or COVID-19)], lipopolysaccharide (LPS), lysophosphatidylcholine (LPC), shear stress, hyperlipidemia and oxidized low-density lipoprotein (oxLDL) (35). ECs execute innate antiviral machinery by inducing expression of several immunomodulatory genes in response to interferons (IFNs) and double-stranded RNA (dsRNA) (36); and ECs have capacities in producing dsRNA protein kinase and activating intrinsic and extrinsic apoptotic pathways for restricting viral dissemination and eliminating viral infection (37). In addition to PAMPs derived from microbes, ECs express caspase 1/inflammasomes (38), one type of PAMPs/DAMPs receptors (PRRs) (2), and sense metabolites-derived conditional DAMPs, as we reported (39, 40), from CVD risk factors such as hyperlipidemia (8, 41), hyperhomocysteinemia (42), uremia toxins associated with CKD (20), and hypoxia (12), which activate ECs and accelerate vascular inflammation (17, 20) and atherosclerosis (43, 44). Mitochondrial reactive oxygen species (mtROS) and proton leak mediate a newly-termed physiological and pathological activation (45–48). Moreover, under stimulation by proatherogenic lipids chronic disease conditions such as hyperlipidemia, ECs have a novel prolonged activation status, as we reported, with four innate immune features, namely, upregulation of EC adhesion molecules (49) and secretion of cytokines and chemokines (50), upregulation of additional DAMP receptors such as CD36, and increased expression of co-signaling receptors and MHC class II molecules (51). Furthermore, the acetylation of histone 3 lysine 14 (H3K14) in genomic regions that encode trained immunity enzymes in LPC-activated human aortic ECs (HAECs) is increased in comparison to the genomic areas that encode for EC activation genes (52). These findings suggest that the acetylation of H3K14 participates in mediating innate immune memory (trained immunity, TI) function of ECs, which are not suppressed by anti-inflammatory and anti-EC activation cytokines IL-35 (53–55) and IL-10 (53, 56). In addition to facilitating inflammatory cell trans-EC migration and immune responses, EC increase the expression of T cell co-stimulation receptors and immune checkpoint receptors/T cell co-inhibition receptors when stimulated by tumor necrosis factor-α (TNFα) and IFNγ (57), suggesting that EC may also play immune tolerogenic function during inflammation *via* reverse signaling of immune checkpoint receptors (31, 58). However, an important question remained whether aortic cells and ECs have various secretomes that are similar to that identified in monocytes and macrophages (32, 59), making cytokine response amplified into cytokine storms. In addition, secretomic gene expression changes have not been extensively examined in aortic ECs and other aortic cells.

Regardless of the significant progress in understanding roles of aortic cells in immune responses modulating the pathogenesis of atherosclerosis, CKD-accelerated vascular inflammation and AAA, a few important questions remained. *First*, whether aorta serves as an immune organ by upregulating the expressions of canonical and non-canonical secretomic genes (SGs) (21, 26) in major metabolic CVD such as atherosclerosis, CKD-accelerated vascular inflammation and AAA; *second*, whether under PAMPs and DAMPs stimulation, vascular ECs and VSMCs are equipped with different secretomic settings; *third*, whether various types of SGs have differential roles in various diseases; and *fourth*, secretomic genes upregulated in diseases and PAMPs and DAMPs stimulation are partially regulated by reactive oxygen species (ROS) regulators and trained immunity regulators (60). Our new transcriptomic results in addressing these issues provide novel insights on the roles of aortic cell and vascular cell secretomes in COVID-19 related virus infection, CVDs, inflammations, transplantation, autoimmune diseases, and cancers.

## Materials and Methods

### Aorta Transcriptomics Data and Database Content

**Figure 1:**
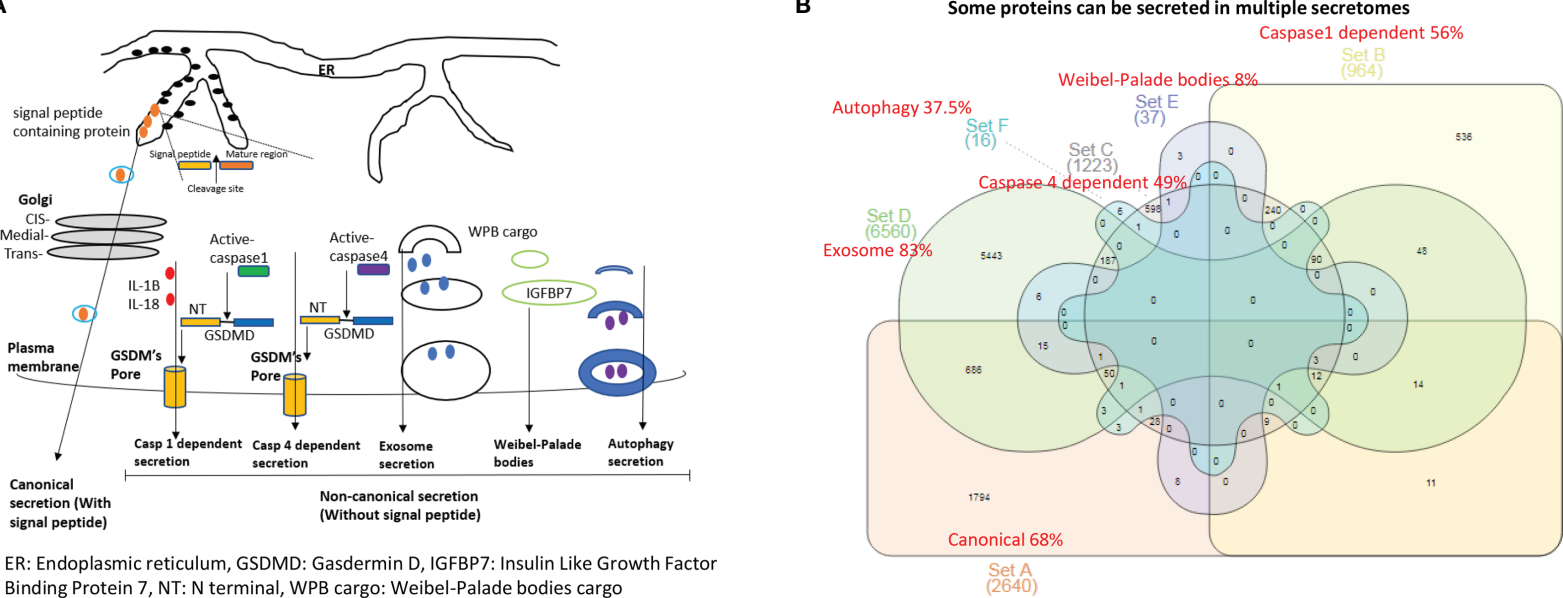
The 53.7% out of 21,306 human protein genes can be classified into six secretomes. **(A)** Six secretomes, canonical (with signal peptide), caspase-1 (secretome mediated via N-terminal gasdermin D protein-formed channel when caspase-1 is activated), caspase-4 (secretome mediated via N-terminal gasdermin D protein-formed protein channel when caspase-4 (humans)/caspase-11 (mice) is activated) and exosomes, are the large secretomes with > 900 proteins, Weibel-Palade bodies (WPB), and autophagy secretome. **(B)** Venn Diagram Analysis was used to classify all the secretory protein genes into the secretome-shared genes and secretome-specific genes in six secretomes, which are ranked from the highest specificity to the lowest as exosome (83%) > canonical (68%) > caspase-1 (56%) > caspase-4 (49%) > autophagy (37.5%) > WPB (8%).

Aorta and endothelial cell transcriptomics datasets were collected from the National Institutes of Health (NIH)–National Center for Biotechnology Information (NCBI)–Gene Expression Omnibus (GEO) (https://www.ncbi.nlm.nih.gov/gds/) and ArrayExpress (https://www.ebi.ac.uk/arrayexpress/) databases and analyzed with online software GEO2R (https://www.ncbi.nlm.nih.gov/geo/geo2r/), namely, atherosclerotic mouse aorta (GSE10000) and CKD affected rat aorta (GSE146638), abdominal aortic aneurysm in aortas of patients (GSE47472), aorta of Ang-II induced aneurysm model (GSE17901), aorta of elastase induced aneurysm (GSE51229), venous endothelial cell from arteriovenous fistula (GSE46126), MERS-CoV infection in human endothelial cells (GSE79218) and Ang-II treated vascular smooth muscle cells (GSE97470). Thus, a total of 8 datasets were analyzed. In addition, we organized six secretomic pathway gene lists from previous publications, namely, canonical secretome (Atlas website (https://www.proteinatlas.org/)), caspase 1-Gasdermin D (caspase 1) secretome (PMID 18329368), caspase 4-Gasdermin D (caspase 4) secretome (PMID28196878), exosome secretome (http://exocarta.org/download), Weibel-Palade bodies (WPB) (PMID 22468712) and autophagy secretome (PMID: 29941450, PMID:30894055, PMID:33363205, PMID: 25988755, PMID: 32332096, PIMD: 31972172) (**Figure 1A**).

### Metascape Analysis

Metascape (https://metascape.org/gp/index.html#/main/step1) was used for enrichment analysis. This website contains the core of most existing gene annotation portals (**Table 1**).

**Table 1:** The most 89 top functional pathways carried out by five secretomes are secretome-specific and are not shared by other secretomes, suggesting that five secretomic mechanisms have different functions.

Table 1A												
	Canonical	Canonical	Canonical	Canonical	Canonical	Caspase1	Caspase1	Caspase1	Caspase4	Caspase4	Exosomes	Exosome
	Specific	Caspase1	Caspase4	Exosomes	WPB	Specific	Caspase4	Exosomes	Specific	Exosomes	Specific	WPB
Actin filament based process	1											
Regulated exocytosis	1			1			1		1			
Hemostasis	1											
Signaling by Receptor Tyrosine Kinases	1											
Organelle localization	1											
Cell morphogenesis involved in differentiation	1											
Regulation of cell adhesion	1				1							
Adaptive immune system	1		1									
Membrane Trafficking	1											
Cellular response to nitrogen compound	1											
DNA conformation change		1										
Cell substrate adherens junction assembly		1										
Regulation of mRNA metabolic process		1										
Detoxification of ROS		1										
Orc1 removal from chromatin		1										
Disease of signal transduction by growth factor receptors and second messengers		1										
Stem cell differentiation		1										
establishment of protein localization to endoplasmic reticulum		1										
Cellular response to abiotic stimulus		1										
actomyosin structure organization		1										
Leukocyte degranulation			1									
Endocytosis			1									
RHO GTPase Effectors			1									
Golgi vesicle transport			1									
positive regulation of organelle organization			1				1					
phagocytosis			1									
Establishment of organelle localization			1									
regulation of leukocyte activation			1									
cytosolic transport			1									

Table 1B												
	Canonical	Canonical	Canonical	Canonical	Canonical	Caspase1	Caspase1	Caspase1	Caspase4	Caspase4	Exosomes	Exosome
	Specific	Caspase1	Caspase4	Exosomes	WPB	Specific	Caspase4	Exosomes	Specific	Exosomes	Specific	WPB
NABA matrisome associated				1						1		
extracellular structure organization				1							1	
NABA core matrisome				1								
humoral immune response				1							1	
complement and coagulation cascades				1								
NABA secreted factors				1								
negative regulation of proteolysis				1								
regulation of IGF transport and uptake by IGFBPs				1								
response to wounding				1								
Cell surface interactions at the vascular wall					1							
Peptidyl tyrosine phosphorylation					1							
negative regulation of supramolecular fiber organization						1						
transcription of the HIV genome						1						
oxidative phosphorylation						1						
CDC5L complex						1						
mRNA catabolic process						1						
chaperone mediated protein transport						1						
positive regulation of viral process						1						
NABA ECM Glycoproteins						1						
Cell-cell communication						1						
Interconversion of nucleotide di-and triphosphates						1						
organic cyclic compound catabolic process							1					
carbon metabolism							1					
protein folding							1					
IL12 mediated signaling pathway							1					
HIF-1 signaling pathway							1					
pentose phosphate metabolism							1					
establishment of protein localization to organelle							1					
cellular response to oxidative stress							1					

Table 1C												
	Canonical	Canonical	Canonical	Canonical	Canonical	Caspase1	Caspase1	Caspase1	Caspase4	Caspase4	Exosomes	Exosome
	Specific	Caspase1	Caspase4	Exosomes	WPB	Specific	Caspase4	Exosomes	Specific	Exosomes	Specific	WPB
Defective EXT2 causes exostoses 2								1				
cell substrate adhesion								1				
VEGFA VEGFR2 signaling pathway								1				
positive regulation of cytokine production								1				
chemotaxis								1			1	
small molecule catabolic process									1			
aromatic compound catabolic process									1			
cofactor metabolic process									1			
oxidation reduction process									1			
alpha amino acid metabolic process									1			
cysteine and methionine metabolism									1			
platelet degranulation									1			
drug catabolic process									1			
purine ribonucleoside metabolic process									1			
myeloid leukocyte activation										1		
positice regulation by host of viral process										1		
regulation of endothelial cell proliferation										1		
carbohydrate derivative catabolic process										1		
intermembrane lipid transfer										1		
fatty acid derivative biodynthetic process										1		
metabolism of vitamins and cofactors										1		
rhythmic process										1		
response to virus										1		
Cytokine-cytokine receptor interaction											1	
defense response to other organism											1	
GPCR ligand binding											1	
PI3K-akt signaling pathway											1	
glycoprotein metabolic process											1	
skeletal system development											1	
aminoglycan metabolic process											1	
response to unfolded protein												1

The signal pathways of the gene list from Figure 1B with 12 groups were analyzed by Metascape (https://metascape.org/gp/index.html#/main/step1).

### Ingenuity Pathway Analysis (IPA)

We utilized Ingenuity Pathway Analysis (IPA, Qiagen, https://www.qiagenbioinformatics.com/products/ingenuity-pathwayanalysis/) to characterize clinical relevance and molecular and cellular functions related to the identified genes in our microarray analysis. Differentially expressed genes were identified and uploaded into IPA for analysis. The core and pathways analysis were used to identify molecular and cellular pathways.

## Results

### Approximately 53.7% out of 21,306 human protein-encoding genes are classified into six secretomes; canonical, caspase 1, caspase 4 and exosomes, are the large secretomes with >900 proteins; and six secretomes are ranked from high to low in specificity as exosome (83%), >canonical (68%), >caspase 1 (56%), >caspase 4 (49%), >autophagy (37.5%), and >WPB (8%).

It remains unknown whether aortic vascular cells such as ECs use canonical secretome (secretory proteins with signal peptide) (21), caspase 1-Gasdermin D (caspase 1) secretome, caspase 4-Gasdermin D (caspase 4) secretome, exosome secretomes (23), Weibel–Palade bodies (WPB), and autophagy secretome. We hypothesized that the expressions of aortic vascular cell canonical secretome, five types of noncanonical secretomes such as caspase 1, caspase 4, exosomes, WPB, and autophagy secretome are differentially modulated in aortic vascular cells in metabolic CVDs and coronavirus infection. To examine this hypothesis, as shown in **Figures 1A, B**, we collected six types of secretomes with a total of 11,440 proteins, namely, canonical secretome (signal peptide-mediated exocytic secretory pathway; 2,640 proteins (21), caspase 1-dependent noncanonical secretome (non-signal peptide-mediated; 964 proteins) (61), caspase 4-dependent noncanonical secretome (1,223 proteins) (62), exosome secretome (6,560 proteins, downloaded from a comprehensive exosome database http://exocarta.org/download) (23), WPB secretome (37 proteins) and autophagy secretome (16 proteins). Of note, most updated human protein-coding genes are 21,306 (63), suggesting that 53.7% of human proteins (11,440 secretory protein genes in the six secretomes out of 21,306 human protein-encoding genes) carry out secretory functions physiologically and/or patho-physiologically.

We performed Venn diagram analysis to determine the overlaps between six types of secretomes. As shown in **Figure 1B**, canonical secretome had 1,794 specific secretomic genes (**SGs**) out of 2,640 SGs (68%); caspase 1 dependent secretome had 536 specific SGs out of 964 SGs (56%); caspase 4 dependent secretome had 598 specific SGs out of 1,223 SGs (49%); exosome secretome had 5,443 specific SGs out of 6,560 (83%); WPB secretome had 3 specific SGs out of 37 SGs (8%); and autophagy secretome had 6 specific SGs out of 16 SGs (37.5%). Our results have demonstrated that some secretory proteins can be secreted *via* different secretomes. Based on their specificities, we ranked six secretomes from high to low in specificities as exosome secretome (83%) > canonical secretome (68%) > caspase 1 secretome (56%) > caspase 4 secretome (49%) > autophagy secretome (37.5%) > WPB secretome (8%).

We then hypothesized that each secretomic protein carries out specific cellular functions. To examine this hypothesis, we analyzed the SGs with the Metascape signaling database (https://metascape.org/gp/index.html#/main/step1). As the top function pathways listed in **Tables 1A–C**, canonical secretome carried out ten specific pathways, namely, actin filament based process, regulated exocytosis (shared with caspase 4 secretome, exosome secretome), hemostasis, signaling by receptor tyrosine kinase, organelle localization, cell morphogenesis involved in differentiation, regulation of cell adhesion (shared with WPB secretome), adaptive immune system (shared with caspase 4 secretome), membrane trafficking, and cellular response to nitrogen compound. Caspase 1 secretome carried out ten specific pathways, namely, negative regulation of supramolecular fiber organization, transcription of HIV genome, oxidative phosphorylation, CDC5L complex (positive regulator of cell cycle G_2_/M progression), mRNA catabolic process, chaperone mediated protein transport, positive regulation of viral process, NABA extracellular matrix (ECM) glycoproteins, cell–cell communications, and interconversion of nucleotide di- and triphosphates. Caspase 4 secretome carried out nine specific pathways, namely, small molecule catabolic process, aromatic compound catabolic process, cofactor metabolic process, oxidation reduction process, alpha amino acid metabolic process, cysteine and methionine metabolism, platelet degranulation, drug catabolic process, and purine ribonucleoside metabolic process. Exosome secretome carried out eight specific pathways, namely, response to virus, cytokine–cytokine receptor interaction, defense system to other organism, G protein-coupled receptor (GPCR) ligand binding, phosphoinositide 3-kinase (PI3K)-protein B kinase (PKB, Akt) signaling pathway, glycoprotein metabolic process, skeletal system development, and aminoglycan metabolic process. Of note, autophagy had 6 specific SGs, for which no specific pathways were found in this analysis. In addition, canonical and caspase 1 secretomes shared 10 specific pathways, canonical and caspase 4 shared ten specific pathways, canonical and exosome secretomes shared ten specific pathways, canonical and WPB secretomes shared 3 specific pathways, caspase 1 and caspase 4 secretomes shared ten specific pathways, caspase 1 and exosome secretomes shared 5 specific pathways, caspase 4 and exosome secretomes shared 10 specific pathways, and exosome and WPB secretomes shared one specific pathway.

Taken together, our results have demonstrated that *first*, 53.7% out of 21,306 human protein-encoding genes in the six secretomes carry out secretory functions physiologically and pathophysiologically; *second*, out of six types of secretomes, canonical, caspase 1, caspase 4 and exosome secretomes are the large secretomes with more than 900 proteins; and *third*, although different types of secretomes share some functions such as regulated exocytosis, regulation of cell adhesion, innate and adaptive immune systems, positive regulation of organelle organization (64), and others, except autophagy secretome, five types of secretomes have specific functional pathways.

### Atherosclerosis, CKD, and AAA Significantly Modulate the Expression of Six Secretomes in Aortas; and MERS-CoV Infection in Human Endothelial Cells and Angiotensin-II Treatment in Vascular Smooth Muscle Cells Also Modulate the Expression of Six Secretomes.

We proposed a new working model that ECs are innate immune cells (3, 32, 65); and more than 20 cytokines have been reported to play roles in mediating EC responses to inflammation in CVD (2, 12, 33, 50, 53, 54, 56, 66–69), immune responses (69), EC-to-mesenchymal transition (70, 71). However, a panoramic view on transcriptomic changes of whole SGs in ECs remained unknown. We hypothesized that SGs in aortic vascular cells have transcriptomic changes in response to PAMPs from viruses, and DAMPs derived from the risk factors of cardiovascular and metabolic diseases such as hyperlipidemia (9, 10, 39, 40, 44, 72), CKD (16, 18–21), and AAA (31).

We and others reported that the Nod-like receptor family 3 (NLRP3) promotes human aortic EC activation (41) induced by oxLDL and LPC (8, 10, 73). The inflammatory cell death (pyroptosis) (9) carried out by caspase 1 (38) canonical, caspase 4 (humans)/caspase 11 (mice) noncanonical inflammasomes (74)-gasdermin D (75) pathway has been reported to mediate EC pyroptosis (inflammatory cell death) (76). Due to their content enriched in proteins, mRNAs and noncoding RNAs, exosome secretomes carry out cell–cell communication and promote tissue regeneration, wound healing, extracellular matrix remodeling, immunomodulation (23), angiogenesis, anti-apoptotic activity and cell migration, proliferation and differentiation (77). In addition, autophagy has unconventional protein secretion (78), which share with extracellular vesicles the molecular mechanisms, namely, with notable crosstalk such as amphisomes and “secretory autophagy” (79). Moreover, ECs have specific WPB secretome (80).

**Figure 2:**
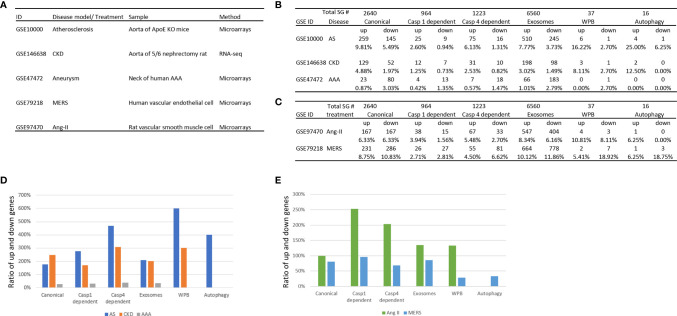
The three major aortic diseases including atherosclerosis, chronic kidney disease (CKD), and abdominal aortic aneurysm (AAA) significantly modulate the expression of six secretomes in aortas **(A–C)**, suggesting that aorta is a new immune and endocrine organ in pathologies. Middle East Respiratory Syndrome Coronavirus (MERS-CoV) infection in human endothelial cells and Angiotensin-II (Ang-II) treatment in vascular smooth muscle cells also modulate the expression of six secretomes **(A–C)**, suggesting that in response to danger associated molecular pattern (DAMPs, Ang-II) pathogens-associated molecular pattern (PAMPs, MERS-CoV) major aortic cell types such as endothelial cells and vascular smooth muscle cells modulate the expression of six secretomes. **(D)** Atherosclerosis and chronic kidney disease modulate the expression of secretomic genes much more than abdominal aortic aneurysm. **(E)** Angiotensin-II upregulates the expression of secretomic genes much more than downregulating the expression of secretomic genes where MERS-CoV infection downregulates more than upregulates the expression of secretomic genes. P < 0.05 |LogFC|>1. LogFC>1 or < -1.

We hypothesized that metabolic CVD such as atherosclerotic mouse aorta and CKD affected rat aorta, MERS-CoV infection in human ECs and AAA in aortas of patients differentially regulate the expression of various types of secretomes. To examine this hypothesis, as shown in **Figure 2A**, we collected microarray and RNA-sequencing data from atherosclerotic apolipoprotein E deficient (ApoE^−/−^) aorta, CKD accelerated vascular inflammation in rat aorta, MERS-CoV infected human ECs and AAA in patients aortas, deposited in the NIH/NCBI-GeoDatasets database (https://www.ncbi.nlm.nih.gov/gds). As shown in **Figures 2B, C**, atherosclerosis upregulated 9.8% and downregulated 5.5% of canonical secretome, upregulated 2.6% and downregulated 0.9% of caspase 1 secretome, upregulated 6.1% and downregulated 1.3% caspase 4 secretome, upregulated 7.8% and downregulated 3.7% of exosome secretome, upregulated 16.2% and downregulated 2.7% of WPB secretome, and upregulated 25% and downregulated 6.2% autophagy secretome. In addition, MERS coronavirus infection in human ECs upregulated 8.8% and downregulated 10.8% of canonical secretome, upregulated 2.7% and downregulated 2.8% of caspase 1 secretome, upregulated 4.5% and downregulated 6.6% caspase 4 secretome, upregulated 10.1% and downregulated 11.9% of exosome secretome, upregulated 5.4% and downregulated 18.9% of WPB secretome, and upregulated 6.3% and downregulated 18.8% autophagy secretome. Moreover, CKD upregulated 4.9% and downregulated 2.0% of canonical secretome, upregulated 1.3% and downregulated 0.7% of caspase 1 secretome, upregulated 2.5% and downregulated 0.8% of caspase 4 secretome, upregulated 3.2% and downregulated 1.5% of exosome secretome, upregulated 8.1% and downregulated 2.7% of WPB secretome, and upregulated 12.5% and downregulated 0% autophagy secretome. Furthermore, AAA upregulated 0.9% and downregulated 3.3% of canonical secretome, upregulated 0.4% and downregulated 1.4% of caspase 1 secretome, upregulated 0.6% and downregulated 1.5% caspase 4 secretome, upregulated 1.0% and downregulated 2.8% of exosome secretome, upregulated 0% and downregulated 2.7% of Weibel–Palade body secretome, and upregulated 0% and downregulated 0% autophagy secretome.

We then hypothesized that upregulation and downregulation of secretomes are different in various diseases. As shown in **Figure 2D**, the ratios of upregulation versus downregulation of canonical secretome, caspase 1 secretome, caspase 4 secretome, exosome secretome, WPB secretome and autophagy secretome in atherosclerosis, CKD, and AAA were high in atherosclerosis and CKD. In contrast, the ratios of upregulation versus downregulation of secretomic genes were low in AAA (**Figure 2C**), which were lower than 0.5 in AAA. In addition, WPB secretomes and autophagy secretome were modulated in atherosclerosis, and CKD much high than that in AAA. The modulation patterns of canonical and exosome secretomes in atherosclerosis, CKD and AAA were similar; and the modulation scales of caspase 4 secretome were bigger than that of caspase 1 secretome in these four diseases.

We then hypothesized that the percentages of modulation (upregulation and downregulation) of each type of secretomic genes are different in various vascular cells. As shown in **Figures 2C, E**, MERS-CoV infection modulated all types of secretomes in human microvascular ECs with upregulation of 231 (8.75%) canonical secretomic genes, 26 (2.71%) caspase 1 secretomic genes, 55 (4.5%) caspase 4 secretomic genes, 664 (10.12%) exosome secretomic genes, 2 (5.41%) WPB secretomic genes and one (6.25%) autophagy secretomic gene, respectively. In addition, MERS infection also downregulated 286 (10.83%) canonical secretomic genes, 27 (2.81%) caspase 1 secretomic genes, 81 (6.62%) caspase 4 secretomic genes, 778 (11.86%) exosome secretomic genes, 7 (18.92%) WPB secretomic genes and 3 autophagy secretomic genes, respectively. Of note, MERS infection downregulated more than upregulated the secretomic genes in MERS-CoV-infected ECs (**Figure 2D**). In comparison, angiotensin-II (Ang-II) treatment of rat VSMCs modulated the expression of all six major secretomes with upregulation of 167 (6.33%) canonical secretomic genes, 38 (3.94%) caspase 1 secretomic genes, 67 (5.48%) caspase 4 secretomic genes, 547 (8.34%) exosome secretomic genes, 4 WPB secretomic genes and one autophagy secretomic gene, respectively. In addition, Ang-II treatment of VSMC downregulated 167 (6.33%) canonical secretomic genes, 15 (1.56%) caspase 1 secretomic genes, 33 (2.7%) caspase 4 secretomic genes, 404 (6.16%) exosome secretomic genes, 3 (8.11%) WPB secretomic genes and 0 autophagy secretomic gene, respectively. The ratios of upregulation versus downregulation of caspase 1 secretomic genes (250%) and that of caspase 4 secretomic genes (200%) were much bigger than that of other secretomic genes (**Figure 2D**), suggesting that significant roles of caspase 1 secretome and caspase 4 secretome in Ang-II treated VSMC inflammatory responses (81). The ratios of upregulation versus downregulation of secretomic genes in Ang-II treated VSMC were much higher than that of MERS-CoV infected human ECs (**Figure 2D**). Of note, due to the differences in experimental conditions and cell types in Ang-II treated VSMC and Ang-II induced AAA, the ratios of upregulation versus downregulation of secretomic genes in Ang-II treated VSMC (>1) and AAA (<1) were different.

Taken together, our data have demonstrated that *first*, atherosclerosis and CKD significantly modulate the expression of six types of secretomes, but AAA modulate the expression of six types of secretomes much less; *second*, the ratios of upregulation versus downregulation of secretomic genes in atherosclerosis and CKD were much higher than that in AAA. In contrast, the ratios of upregulation versus downregulation of secretomic genes were low in AAA, suggesting that atherosclerosis and CKD-aorta need more general secretomic functions than AAA, but AAA require more focused secretomic functions than atherosclerosis and CKD-aorta; *third*, in addition to global secretomic genes analysis in aorta, we also examined the expressions of vascular cell secretomic genes. Ang-II treatment of aortic VSMCs and MERS-CoV-infection of ECs significantly modulate the expression of SGs. The ratios of upregulation versus downregulation of caspase 1 SGs and that of caspase 4 SGs are much bigger than that of other SGs in Ang-II treated VSMCs; and the ratios of upregulation versus downregulation of SGs in Ang-II treated VSMC are bigger than that of MERS-infected ECs, and *fourth*, canonical secretome, exosome secretome, WPB secretome, and autophagy secretome are general pathology secretomes except WPB secretome in atherosclerosis but caspase 1 secretome and caspase 4 secretome are DAMP receptors-focused and inflammatory secretomes such as chronic inflammation, namely, atherosclerosis, CKD, and Ang-II treated VSMC.

### Atherosclerotic Aortas Upregulate T Cell and B Cell Adaptive Immune Secretomic Genes; CKD Aortas Upregulate Secretomic Genes for Cardiac Hypertrophy, Hepatic Fibrosis and Senescence; and AAA Aortas Upregulate Secretomic Genes for Neuromuscular Signaling, Protein Catabolic Process and Fcγ Receptor-Mediated Phagocytosis

**Figure 3:**
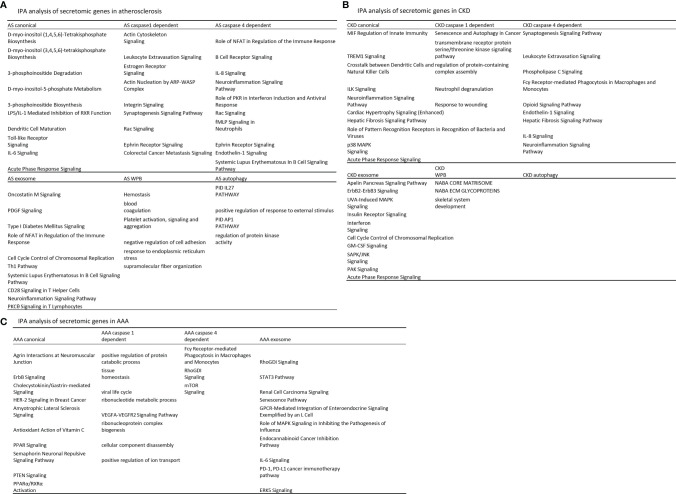
Atherosclerosis (AS) aortas upregulate T and B cell immune secretomic genes (SGs); chronic kidney disease (CKD) aortas upregulate SGs for cardiac hypertrophy and hepatic fibrosis; and abdominal aortic aneurysm (AAA) aortas upregulate SGs for neuromuscular signaling and protein catabolism. **(A)** Ingenuity Pathway Analysis (IPA) analysis of upregulated genes in Fig. 2B. Atherosclerotic aortas upregulate expression of T cell and B cell adaptive immune secretomic genes; **(B)** chronic kidney disease (CKD) aortas upregulate expression of secretomic genes for cardiac hypertrophy, hepatic fibrosis, and senescence; and **(C)** Aortic aneurysm aortas upregulate expression of secretomic genes for neuromuscular signaling, protein catabolic process, and immunoglobulin Fc gamma (g) receptor-mediated phagocytosis. Z score>1.

We hypothesized that each type of secretomic genes play different roles in diseases. To examine this hypothesis, we analyzed the functional signaling pathways in differentially modulated secretomic genes in each disease setting. As shown in **Figure 3A**, for example, canonical secretomic genes in atherosclerotic aortas had metabolic and inflammatory functions, namely, D-myo-inositol (1,4,5,6)-tetrakisphosphate biosynthesis, D-myo-inositol (3,4,5,6)-tetrakisphosphate biosynthesis, 3-phosphoinsitide degradation, D-myo-inositol 5-phosphate metabolism, 3-phosphoinsitide biosynthesis, lipopolysaccharide (LPS)/IL-1 mediated inhibition of retinoid X receptor (RXR) function, dendritic cell maturation, Toll-like receptor signaling, IL-6 signaling, and acute phase response signaling. Caspase 1-GSDMD secretomic genes in atherosclerosis had various pathophysiological functions, namely, actin cytoskeleton signaling, leukocyte extravasation signaling, estrogen receptor signaling, actin nucleation by Actin Related Protein 2/3 complex (ARP)-Wiskott–Aldrich syndrome protein (WASP) complex, integrin signaling, synaptogenesis signaling, Rac signaling, Ephrin receptor signaling, and colorectal cancer metastasis signaling. Caspase 4-GSDMD secretomic genes in atherosclerosis had various pathophysiological functions with B cell signaling, namely, role of nuclear factor of activated T cells (NFAT) in regulation of immune response, B cell receptor signaling, IL-8 signaling, neuroinflammation signaling, role of protein kinase R (PKR) in interferon induction and antiviral response, Rac signaling, fMLP signaling in neutrophils, Ephrin receptor signaling, endothelin 1 signaling, and systemic lupus erythematosus in B cell signaling. Exosome secretomic genes in atherosclerosis had various pathophysiological functions with T cell signaling, namely, oncostatin M signaling, platelet derived growth factor (PDGF) signaling, type-I diabetes signaling (autoimmune), role of NFAT in regulation of immune response (82), cell cycle control of chromosomal replication, type 1 T helper cell (Th1) pathway, systemic lupus erythematosus in B cell signaling, CD28 signaling in T helper cells, neuroinflammation signaling, and PKCθ signaling in T cells. Weibel–Palade body SGs in atherosclerosis had coagulation and platelet activation functions, namely, hemostasis, blood coagulation, platelet activation, negative regulation of cell adhesion, response to endoplasmic reticulum stress, and supramolecular fiber organization. Autophagy SGs in atherosclerosis had functions such as pathway interaction database (PID) (83) IL27 pathway (https://maayanlab.cloud/Harmonizome/dataset/PID+Pathways), positive regulation of response to external stimulus, PID AP1 pathway, and regulation of protein kinase activity. These results have demonstrated that atherosclerotic aorta cells modulate different SGs for different functions with cell-type specificities. For example, canonical secretomic genes for metabolic reprogramming, inflammation; caspase 1-secretomic genes for cytoskeletal signaling, leukocyte extravasation signaling; caspase 4 SGs for B cell signaling and NFAT-related cytokine signaling; exosome SGs for T cell signaling and autoimmune signaling; Weibel–Palade body SGs for blood coagulation and platelet activation; and autophagy SGs for positive response to external stimulus and regulation of protein kinase activity.

As shown in **Figure 3B**, canonical SGs in CKD aortas had functional pathways, namely, MIF regulation of innate immunity, triggering receptor expressed on myeloid cells 1 (TREM1) signaling, crosstalk between dendritic cells and natural killer cells, integrin-linked kinase (ILK) signaling, neuroinflammation signaling, cardiac hypertrophy signaling, hepatic fibrosis signaling, role of pattern recognition receptors in recognition of bacteria and viruses, p38 mitogen-activated protein kinase (MARK) signaling, and acute phase response signaling. Caspase 1 SGs in CKD had functional pathways, namely, senescence and autophagy, transmembrane receptor protein serine/threonine kinase signaling, regulation of protein containing complex assembly, neutrophil degranulation, and response to wounding. Caspase 4 SGs in CKD had functional pathways, namely, synaptogenesis signaling, leukocyte extravasation, phospholipase C signaling, Fcγ receptor-mediated phagocytosis in macrophages and monocytes, opioid signaling, endothelin 1 signaling, hepatic fibrosis signaling, IL-8 signaling, and neuroinflammation signaling. Exosome SGs in CKD had functional pathways, namely, apelin pancreas signaling, receptor tyrosine-protein kinase ErbB2 (human epithelial growth factor receptor 2, HER-2)–receptor tyrosine-protein kinase erbB3 (HER-3) signaling, UVA induced MAPK signaling, insulin receptor signaling, interferon signaling, cell cycle control of chromosomal replication, granulocyte–macrophage colony stimulation factor (GM-CSF) signaling, stress activated protein kinase (SAPK)/Jun amino-terminal kinase (JNK) signaling, serine/threonine protein kinase family (PAK) signaling, and acute phase response signaling. Weibel–Palate body SGs had NABA core matrisome, NABA ECM glycoproteins (84), and skeletal system development. These results have demonstrated that CKD modulate canonical SGs for innate immunity and inflammation; caspase 1-GSDMD SGs for senescence and response to wounding; caspase 4-GSDMD SGs for leukocyte extravasation, inflammation, and fibrosis; exosome SGs for MAPK signaling, insulin receptor signaling; Weibel–Palade body SGs for NABA complexes and skeletal system. CKD did not significantly modulate autophagy SGs.

As shown in **Figure 3C**, canonical SGs in AAA aortas had functional pathways, namely, agrin interactions in neuromuscular junction, ErbB signaling, cholecystokinin/gastrin-mediated signaling, HER-2 signaling in breast cancer, amyotrophic lateral sclerosis signaling, antioxidant action of vitamin C, peroxisome proliferators activated receptors (PPAR) signaling, semaphorin neuronal repulsive signaling, phosphatase and tensin homolog deleted on chromosome 10 (PTEN) signaling and PPARa/RXRa activation. Caspase 1 SGs in AAA aortas had functional pathways, namely, positive regulation of protein catabolic process, tissue homeostasis, viral life cycle, ribonucleotide metabolic process, vascular endothelial growth factor a (VEGFA)-VEGFR2 signaling, ribonucleoprotein complex biogenesis, cellular component disassembly, and positive regulation of ion transport. Caspase 4 SGs in AAA had functional pathways, namely, Fcγ receptor-mediated phagocytosis in macrophages and monocytes, Rho (a small GTPase protein) GDP-Dissociation Inhibitors (RhoGDI) signaling and mTOR signaling. Exosome SGs in AAA aortas had functional pathways, namely, RhoGDI signaling, Signal transducer and activator of transcription 3 (STAT3) pathway, renal carcinoma signaling, senescence pathway, G protein-coupled receptor (GPCR)-mediated integration of enteroendocrine signaling, role of MAPK signaling inhibiting the pathogenesis of influenza, endocannabinoid cancer inhibition pathway, IL-6 signaling, programmed death-1 (PD-1), programmed death ligand 1 (PD-L1) cancer immunotherapy, and extracellular signal-regulated kinase 5 (ERK5) signaling. Autophagy SGs had no functional pathways in AAA aorta.

Taken together, our results have demonstrated for the first time that *first*, aortic cells in three cardiovascular metabolic diseases such as atherosclerosis, CKD, and AAA modulate different SGs for various pathophysiological functions and cell-type specific functions (B cell signaling in caspase 4-GSDMD secretome and T cell signaling in exosome secretome); *second*, cell-type specific functions of caspase 4-GSDMD secretome is atherosclerotic aortas-specific, which do not appear in CKD and AAA aortas; *third*, exosome secretome carry out T cell signaling function not only in atherosclerotic aortas but also in AAA aortas STAT3 pathway (85) and PD-1, PD-L1 immune checkpoint pathway (26, 57); *fourth*, autophagy secretome has atherosclerotic functions of IL-27 and positive regulation of response to external stimulus and does not play significant roles in CKD and AAA aortas; and *fifth*, WPB secretome has blood coagulation and platelet activation functions in atherosclerotic aortas but has extracellular matrix functions (NABA complexes) (84) in CKD aortas.

### Canonical Secretome in Atherosclerotic Aortas Upregulate Pattern Recognition Receptors and Leukocyte Extravasation at 6 Weeks, T Cell Exhaustion and Fibrosis at 32 and 78 Weeks; Caspase 1, and Caspase 4, Secretomes Have no Functions at 6 Weeks, but Have Functions of Cancer Metastasis, and NFAT Signaling at 32 and 78 Weeks, Respectively; and Exosome Secretome Has eNOS and Calcium Signaling at 6 Weeks but GF-CSF and NFAT Signaling at 32 and 78 Weeks

We then hypothesized that atherosclerotic aortas in different time courses differentially modulate the expression of secretomic genes. To test this hypothesis, we collected time courses microarray datasets of atherosclerotic aortas at high fat feeding for 6, 32, and 78 weeks, respectively, in **Table 2**. Canonical secretomic genes at 6-week atherosclerotic aorta had functional pathways, namely, reelin signaling in neurons, role of pattern recognition receptors in recognition of bacteria and viruses, glycoprotein VI (GP6) signaling, estrogen receptor signaling, role of interferon-induced dsRNA-dependent protein kinase (PKR) in interferon induction and antiviral response, factors promoting cardiogenesis, leukocyte extravasation signaling, osteoarthritis pathway, hepatic fibrosis signaling, and acute phase response signaling. Canonical secretomic genes at 32 weeks atherosclerotic aorta had functional pathways, namely, chondroitin sulfate degradation, ubiquitin-like modifier FAT10 cancer signaling, T cell exhaustion signaling, IL-6 signaling, Toll-like receptor signaling, hepatic fibrosis signaling, role of IL-17F in allergic inflammatory airway disease, neuroinflammation signaling, TREM1 signaling, and acute phase response signaling. Canonical secretomic genes at 78 weeks atherosclerotic aorta had functional pathways, namely, five metabolic signaling pathways such as D-myo-inositol (1,4,5,6)-tetrakisphosphate biosynthesis, D-myo-inositol (3,4,5,6)-tetrakisphosphate biosynthesis, 3-phosphinositide degradation, D-myo-inositol-5-phosphate biosynthesis metabolism, 3-phosphinositide biosynthesis, and five inflammatory signaling pathways such as LPS/IL-1 mediated inhibition of RXR function, dendritic cell maturation, Toll-like receptor signaling, IL-6 signaling and acute phase response signaling. These results have demonstrated that *first*, atherosclerotic aorta cells differentially modulate the expression of canonical secretomic genes in the three time points; *second*, at 6 weeks atherosclerotic aortas have more role of pattern recognition receptors, interferon induction and leukocyte extravasation signaling; 32 weeks atherosclerotic aortas have more T cell exhaustion, IL-6 signaling and neuroinflammation signaling; 78 weeks atherosclerotic aortas have more metabolic reprogramming, LPS/IL-1 inhibition of RXR function, dendritic cell maturation, and Toll-like receptor (TLR) signaling.

**Table 2:** Aortic secretomes are differentially modulated in different stages of atherosclerotic progression.

	6 weeks	32 weeks	78 weeks
Canonical	Reelin Signaling in Neurons	Chondroitin Sulfate Degradation (Metazoa)	D-myo-inositol (1,4,5,6)-Tetrakisphosphate Biosynthesis
	Role of Pattern Recognition Receptors in Recognition of Bacteria and Viruses	FAT10 Cancer Signaling Pathway	D-myo-inositol (3,4,5,6)-tetrakisphosphate Biosynthesis
	GP6 Signaling Pathway	T Cell Exhaustion Signaling Pathway	3-phosphoinositide Degradation
	Estrogen Receptor Signaling	IL-6 Signaling	D-myo-inositol-5-phosphate Metabolism
	Role of PKR in Interferon Induction and Antiviral Response	Toll-like Receptor Signaling	3-phosphoinositide Biosynthesis
	Factors Promoting Cardiogenesis in Vertebrates	Hepatic Fibrosis Signaling Pathway	LPS/IL-1 Mediated Inhibition of RXR Function
	Leukocyte Extravasation Signaling	Role of IL-17F in Allergic Inflammatory Airway Diseases	Dendritic Cell Maturation
	Osteoarthritis Pathway	Neuroinflammation Signaling Pathway	Toll-like Receptor Signaling
	Hepatic Fibrosis Signaling Pathway	TREM1 Signaling	IL-6 Signaling
	Acute Phase Response Signaling	Acute Phase Response Signaling	Acute Phase Response Signaling
Caspase 1		Integrin Signaling	Actin Nucleation by ARP-WASP Complex
		Estrogen Receptor Signaling	Integrin Signaling
		Colorectal Cancer Metastasis Signaling	Synaptogenesis Signaling Pathway
			Rac Signaling
			Ephrin Receptor Signaling
			Colorectal Cancer Metastasis Signaling
Caspase 4		ERK/MAPK Signaling	Role of NFAT in Regulation of the Immune Response
		IL-8 Signaling	B Cell Receptor Signaling
		Neuroinflammation Signaling Pathway	IL-8 Signaling
		Synaptogenesis Signaling Pathway	Neuroinflammation Signaling Pathway
		Fc Epsilon RI Signaling	Role of PKR in Interferon Induction and Antiviral Response
		Role of PKR in Interferon Induction and Antiviral Response	Rac Signaling
		B Cell Receptor Signaling	fMLP Signaling in Neutrophils
		Endothelin-1 Signaling	Ephrin Receptor Signaling
		HOTAIR Regulatory Pathway	Endothelin-1 Signaling
		Systemic Lupus Erythematosus In B Cell Signaling Pathway	Systemic Lupus Erythematosus In B Cell Signaling Pathway
Exosome	Reelin Signaling in Neurons	GM-CSF Signaling	Oncostatin M Signaling
	eNOS Signaling	Role of NFAT in Regulation of the Immune Response	PDGF Signaling
	Cholecystokinin/Gastrin-mediated Signaling	FAT10 Cancer Signaling Pathway	Type I Diabetes Mellitus Signaling
	Cardiac Hypertrophy Signaling	Cell Cycle Control of Chromosomal Replication	Role of NFAT in Regulation of the Immune Response
	Melatonin Signaling	3-phosphoinositide Biosynthesis	Cell Cycle Control of Chromosomal Replication
	Calcium Signaling	PDGF Signaling	Th1 Pathway
	Synaptic Long Term Depression	IL-8 Signaling	Systemic Lupus Erythematosus In B Cell Signaling Pathway
	Apelin Cardiomyocyte Signaling Pathway	TREM1 Signaling	CD28 Signaling in T Helper Cells
	Cardiac Hypertrophy Signaling (Enhanced)	PKCθ Signaling in T Lymphocytes	Neuroinflammation Signaling Pathway
	Endothelin-1 Signaling	Neuroinflammation Signaling Pathway	PKCθ Signaling in T Lymphocytes

IPA analysis of modulated genes in different time course (6 weeks, 32 weeks, and 78 weeks) of aorta in atherosclerosis (GSE10000). Z score>1.

Caspase 1-GSDMD SGs at 6 weeks atherosclerotic aortas had no functional pathways. However, at 32 weeks had three functional pathways, namely, integrin signaling, estrogen receptor signaling, and colorectal cancer metastasis signaling and at 78 weeks atherosclerotic aortas had six functional pathways, namely actin nucleation by ARP–WASP complex, integrin signaling, synaptogenesis signaling, Rac signaling, ephrin receptor signaling, and colorectal cancer metastasis signaling. These results have demonstrated that *first*, at as early as 6 weeks, atherosclerotic aortas have no functional caspase 1-GSDMD secretomic functions; *second*, starting from 32 to 78 weeks, atherosclerotic aortas have two major functional pathways, namely, integrin signaling and colorectal cancer metastasis signaling; *third*, estrogen receptor signaling only appears in 32 weeks atherosclerotic aortas whereas four functional pathways appear only in 78 weeks atherosclerotic aortas, namely, actin nucleation by ARP–WASP complex, synaptogenesis signaling, Rac signaling, and ephrin receptor signaling.

Caspase 4 SGs at 6 weeks atherosclerotic aortas had no functional pathways, however, at 32 weeks had functional pathways, namely, ERK/MAPK signaling, IL-8 signaling, neuroinflammation, synaptogenesis, Fc epsilon RI, role of PKR in interferon induction and antiviral response, B cell receptor signaling, endothelin-1 signaling, HOTAIR (a long non-coding RNA) signaling, and systemic lupus erythematosus in B cell signaling. Caspase 4 SGs at 78 weeks atherosclerotic aortas had functional pathways, namely, role of NFAT in regulation of immune response, B cell receptor signaling, IL-8 signaling, neuroinflammation, role of PKR in interferon induction and antiviral response, Rac (a member of small GTPase family) signaling, formyl peptide receptor 1 (fMLP) signaling in neutrophils, ephrin receptor signaling, endothelin-1 signaling, and systemic lupus erythematosus in B cell signaling. These results have demonstrated that *first*, at as early as 6 weeks, atherosclerotic aortas have no functional caspase 4-GSDMD secretomic functions; *second*, starting from 32 to 78 weeks, atherosclerotic aortas have six functional pathways such as IL-8 signaling, neuroinflammation, B cell receptor signaling, endothelin-1 signaling, role of PKR in interferon induction and antiviral response and systemic lupus erythematosus in B cell signaling; *third*, four functional pathways appear only at 32 weeks atherosclerotic aortas, namely, ERK/MAPK, synaptogenesis, Fc epsilon RI, HOTAIR regulatory pathway whereas four other functional pathways appear only at 78 weeks atherosclerotic aortas such as role of NFAT in regulation of immune response, Rac signaling, fMLP signaling in neutrophils, and ephrin receptor signaling.

Exosome SGs at 6 weeks atherosclerotic aortas had functional pathways, namely, Reelin (a large, secreted glycoprotein for correct neuronal positioning) signaling, EC nitric oxide synthase (eNOS) signaling, cholecystokinin/gastrin-mediated signaling, cardiac hypertrophy signaling, melatonin signaling, calcium signaling, synaptic long-term depression, apelin cardiomyocyte signaling, cardiac hypertrophy signaling and endothelin-1 signaling. Exosome SGs at 32 weeks atherosclerotic aortas had functional pathways, namely, granulocyte–macrophage colony stimulating factor (GM-CSF) signaling, role of NFAT in regulation of immune response, HLA-F-adjacent transcript 10 (FAT10, an ubiquitin-like modifier) cancer signaling, cell cycle control of chromosomal replication, 3-phophoinositide biosynthesis, PDGF signaling, IL-8 signaling, TREM1 signaling, protein kinase Cθ (PKCθ) signaling in T cells, and neuroinflammation signaling. Exosome SGs at 78 weeks atherosclerotic aortas had functional pathways, namely, oncostatin M signaling, PDGF signaling, type-I diabetes signaling, role of NFAT in regulation of immune response, cell cycle control of chromosomal replication, Th1 pathway, systemic lupus erythematosus in B cell signaling, CD28 signaling in T helper cells, neuroinflammation signaling and PKCθ signaling in T cells.

These results have demonstrated that *first*, exosome SGs at 6 weeks atherosclerotic aortas have pathophysiological functional pathways such as eNOS, cardiac hypertrophy, endothelin-1 signaling; *second*, starting from 32 to 78 weeks, exosome SGs in atherosclerotic aortas have five functional pathways in common, namely, role of NFAT in regulation of immune response, cell cycle control of chromosomal replication, PDGF signaling, neuroinflammation signaling and PKCθ signaling in T cells; *third*, at 32 weeks exosome SGs in atherosclerotic aortas have several specific functional pathways, namely, GM-CSF, FAT10 cancer signaling, 3-phosphoinositide biosynthesis, IL-8, and TREM1 signaling; whereas at 78 weeks exosome SGs in atherosclerotic aortas have five specific functional pathways, namely, oncostatin M, type-I diabetes, Th1 pathway, systemic lupus erythematosus in B cell signaling, and CD28 signaling in T helper cells.

### Ang II-Induced AAA, Canonical, Caspase 4, and Exosome Secretomes Have Two Peaks of High (Day 7)-Low (Day 14)-High (Day 28) Expression Patterns; Caspase 1, WPB, and Autophagy Secretomes Are Only Functional at Day 7; and the Early Secretomes May Function as Drivers for Trained Immunity and Sustained Inflammation

**Figure 4:**
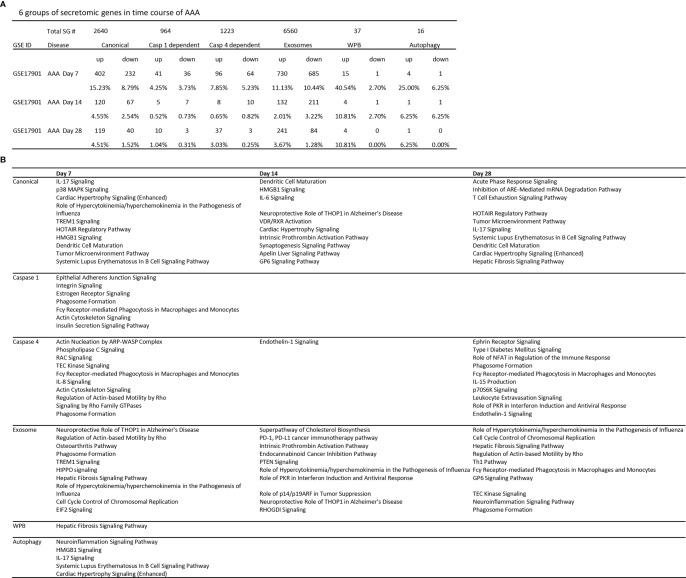
Ang-II induced AAA, canonical, caspase 4, and exosome SGs in aortas have two expression peaks of high (Day 7)-low (Day 14)-high (Day 28) patterns suggesting the evidence of trained immunity response. **(A)** Canonical, caspase 4-GSDMD, and exosome pathways have two peak (three-phase) of high (Day 7)-low (Day 14)-high (Day 28) patterns; and caspase 1-GSDMD, Weibel-Palade body, and autophagy secretomes are only functional at the Day 7 AAA aortas. P < 0.05, LogFC>1 or < -1. **(B)** The signal pathways of secretomic genes are different in AAA progression. Z score>1.

We then hypothesized that abdominal aortic aneurysm aortas in different time courses differentially modulate the expression of secretomic genes. To test this hypothesis, we collected time courses microarray datasets of aneurysm aortas at days 7, 14, and 28. As shown in **Figure 4A**, at day 7, AAA aortas upregulated 402 canonical SGs, 41 caspase 1 SGs, 96 caspase 4 SGs, 730 exosome SGs, 15 WPB SGs and 4 autophagy SGs. At day 14, AAA aortas upregulated 120 canonical SGs, 5 caspase 1 SGs, 8 caspase 4 SGs, 132 exosome SGs, 4 WPB SGs, and 1 autophagy SG. At day 28, AAA aortas upregulated 119 canonical SGs, 10 caspase 1 SGs, 37 caspase 4 SGs, 241 exosome SGs, 4 WPB SGs, and 1 autophagy SG.

We then examined AAA modulated secretomic gene functions by performing Ingenuity Pathway Analysis (IPA). As shown in **Figure 4B**, canonical secretomic genes in day 7 AAA aortas had functional pathways, namely, IL-17 signaling, p38 MAPK signaling, cardiac hypertrophy signaling, role of hypercytokinemia/hyperchemokinemia in the pathogenesis of influenza, TREM1 signaling, HOTAIR pathway, high mobility group box 1 (HMGB1) signaling, dendritic cell maturation, tumor microenvironment pathway, and systemic lupus erythematosus in B cell signaling. Canonical secretomic genes in day 14 AAA aortas had functional pathways, namely, dendritic cell maturation, HMGB1 signaling, IL-6 signaling, neuroprotective role of THOP1 in Alzheimer’s disease, vitamin D receptor (VDR)/RXR activation, cardiac hypertrophy signaling, intrinsic prothrombin activation pathway, synaptogenesis signaling, apelin liver signaling, and GP6 signaling. Canonical secretomic genes in day 28 AAA aortas had functional pathways, namely, acute phase response signaling, inhibition of AU-rich elements (AREs)-mediated mRNA degradation, T cell exhaustion signaling, HOTAIR pathway, tumor microenvironment pathway, IL-17 signaling, systemic lupus erythematosus in B cell signaling, dendritic cell maturation, cardiac hypertrophy signaling, and hepatic fibrosis signaling. These results have demonstrated that *first*, each time point, AAA aortas have their own specific canonical secretomic signaling pathways; *second*, two pathways are shared in all three time points, namely, cardiac hypertrophy signaling, dendritic cell maturation; one pathway is shared in the day 7 AAA aortas and the day 14 AAA aortas, HMGB1; whereas as many as four pathways are shared by the day 7 AAA aortas and the day 28 AAA aortas, namely, HOTAIR pathway, tumor microenvironment pathway, IL-17 signaling and systemic lupus erythematosus in B cell signaling; and *third*, these findings suggest that AAA aortas have two-peak signaling processes in the pathogenesis of AAA.

Caspase 1 SGs at day 7 AAA aortas had functional pathways, namely, epithelial adherens junction signaling, integrin signaling, estrogen receptor signaling, phagosome formation, Fcγ receptor-mediated phagocytosis in macrophages and monocytes, actin cytoskeleton signaling, and insulin secretion signaling. However, caspase 1 SGs at the day 14 AAA aortas and the day 28 AAA aortas have no significant functional pathways.

Caspase 4 SGs at the day 7 AAA aortas had functional pathways, namely, actin nucleation by ARP-WASP complex, phospholipase C signaling, RAC signaling, TEK tyrosine kinase signaling, Fcγ receptor-mediated phagocytosis in macrophages and monocytes, IL-8 signaling, actin cytoskeleton signaling, regulation of actin-based motility by Rho, signaling by Rho family GTPase, and phagosome formation. Caspase 4 SGs at the day 14 AAA aortas had functional pathways of endothelin-1 signaling. Caspase 4 SGs at the day 28 AAA aortas had functional pathways, namely, ephrin receptor signaling, type-1 diabetes signaling, role of NFAT in regulation of immune response, phagosome formation, Fcγ receptor-mediated phagocytosis in macrophages and monocytes, IL-15 signaling, p70S6K signaling, leukocyte extravasation signaling, role of PKR in interferon induction and antiviral response, and endothelin-1 signaling. These results have demonstrated that *first*, once again, caspase 4 SGs at the days 7 and 28 but not day 14 AAA aortas have two-peak signaling processes in the pathogenesis of AAA; *second*, the caspase 4 SGs in the day 14 AAA aortas have only one functional pathway; and *third*, the caspase 4 SGs in the day 7 and day 28 AAA aortas share two functional pathways, namely, Fcγ receptor-mediated phagocytosis in macrophages and monocytes and phagosome formation.

Exosome SGs at the day 7 AAA aortas had functional pathways, namely, neuroprotective role of THOP1 in Alzheimer’s disease, regulation of actin-based motility by Rho, osteoarthritis pathway, phagosome formation, TREM1 signaling, HIPPO (an evolutionarily conserved serine/threonine kinase) signaling, hepatic fibrosis signaling, role of hypercytokinemia/hyperchemokinemia in pathogenesis of influenza, cell cycle control of chromosomal replication, and EIF2 signaling. Exosome SGs at the day 14 AAA aortas had functional pathways, namely, superpathway of cholesterol biosynthesis, PD-1, PD-L1 cancer immunotherapy, intrinsic prothrombin activation, endocannabinoid cancer inhibition pathway, PTEN signaling, role of hypercytokinemia/hyperchemokinemia in pathogenesis of influenza, role of PKR interferon induction and antiviral response, role of p14/p19ARF in tumor suppression, neuroprotective role of THOP1 in Alzheimer’s disease, and RHOGDI signaling. Exosome SGs at the day 14 AAA aortas had functional pathways, namely, role of hypercytokinemia/hyperchemokinemia in pathogenesis of influenza, cell cycle control of chromosomal replication, hepatic fibrosis signaling, regulation of actin-based motility by Rho, Th1 pathway, Fcγ (antibody Fc fragment) receptor-mediated phagocytosis in macrophages and monocytes, GP6 signaling, TEC kinase signaling, neuroinflammation signaling and phagosome formation. These results have demonstrated that *first*, at all three time points one functional pathway, role of hypercytokinemia/hyperchemokinemia in pathogenesis of influenza, is shared; *second*, four functional pathways are shared by the day 7 AAA aortas and day 28 AAA aortas, namely, phagosome formation, regulation of actin-based motility by Rho, cell cycle control of chromosomal replication, and hepatic fibrosis signaling; *third*, one functional pathway of neuroprotective role of THOP1 in Alzheimer’s disease is shared by the day 7 and day 14 AAA aortas; and *fourth*, exosome SGs in AAA aortas have time point-specific functions.

WPB SGs at the day 7 AAA aortas had one functional pathway, hepatic fibrosis signaling. Autophagy SGs at the day 7 AAA aortas had five functional pathways, namely, neuroinflammation signaling, HMGB1 signaling, IL-17 signaling, systemic lupus erythematosus in B cell signaling, cardiac hypertrophy signaling.

Taken together, our results have demonstrated that *first*, among six secretomic pathways in Ang-II induced abdominal aortic aneurysm aortas, canonical, caspase 4, and exosome pathways all have two peak (three-phase) of high (day 7)-low (day 14)-high (day 28) expression patterns; *second*, caspase 1, WPB and autophagy secretomes are only function at the day 7; and *third*, the early secretomes may function as drivers for trained immunity (52, 59, 65) and sustained inflammation (51).

### Elastase Induced AAA Have More Inflammatory and Immune Pathways Than That of Ang-II Induced AAA

**Figure 5:**
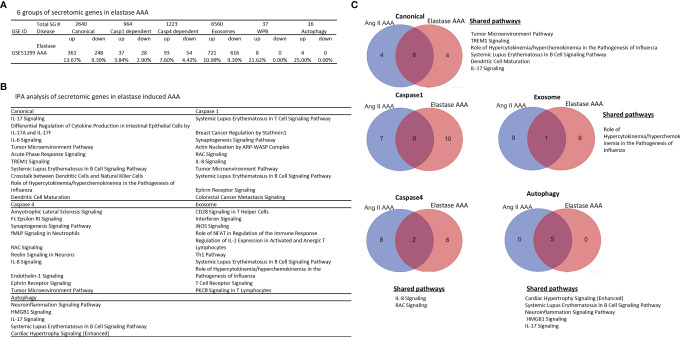
Elastase induced AAA aortas have more inflammatory/immune pathways than that of Ang-II induced AAA aortas. **(A)** The signal pathways of secretomic genes are different in AAA progression. P < 0.05, |LogFC|>1. **(B)** IPA analysis of elastase induced AAA (GSE51229) in each secretory pathway. Z score>1. **(C)** The Venn diagram of upregulated pathways in Day 7 Ang II induced AAA and elastase induced AAA. Z score>1 .

Ang-II induced, elastase induced, and calcium chloride/phosphate induced AAA are three commonly used mouse models (86) in addition to a few surgical mouse models (87) for the induction of AAA. We then hypothesized that elastase induced AAA aortas differentially modulate the expression of secretomic genes from that of Ang-II AAA aortas. To test this hypothesis, we collected elastase induced AAA microarray datasets (GSE51299) (**Figure 5A**). Of note, we could not find microarray datasets for calcium chloride/phosphate-induced AAA for our analyses. Of note, the data in **Figure 4A** showed that Ang-II induced AAA (28 days) upregulated 4.51% canonical secretome, 1.04% caspase 1 secretome, 3.03% caspase 4 secretome, 3.67% exosome secretome, 10.81% WPB secretome, and 6.25% autophagy secretome, respectively. In contrast, in **Figure 5A**, elastase induced AAA upregulated 13.67% canonical SGs (3.03 folds higher than that of Ang-II induced AAA), 3.84% caspase 1 SGs (3.69 folds higher than that of Ang-II induced AAA), 7.60% caspase 4 SGs (2.51 folds higher than that of Ang-II induced AAA), 10.99% exosome SGs (2.99 folds higher than that of Ang-II induced AAA), 21.62% WPB SGs (2.0 folds higher than that of Ang-II induced AAA) and 25.0% autophagy SGs (4.0 folds higher than that of Ang-II induced AAA), respectively, suggesting that elastase induced AAA upregulations of SGs were much more that of Ang-II induced AAA; and that elastase induced AAA can be much more inflammatory than that of Ang-II induced AAA. Of note, the results need to be further consolidated with additional experiments with side-by-side two model comparisons. As shown in **Figure 5B**, canonical SGs in elastase induced AAA aortas had functional pathways of IL-17 signaling, differential regulation of cytokine production by IL-17A and IL-17F, IL-6 signaling, tumor microenvironment pathway, acute phase response, TREM1 signaling, systemic lupus erythematosus in B cell signaling, crosstalk between dendritic cells and natural killer cells, role of hypercytokinemia/hyperchemokinemia, and dendritic cell maturation. Caspase 1-GSDMD SGs in elastase induced AAA aortas had functional pathways, namely, systemic lupus erythematosus in T cell signaling, breast cancer regulation by strathmin1, synaptogenesis signaling, actin nucleation by ARP-WASP complex, RAC signaling, IL-8 signaling, tumor microenvironment pathway, systemic lupus erythematosus in B cell signaling, ephrin receptor signaling, and colorectal cancer metastasis signaling. Caspase 4 SGs in elastase induced AAA aortas had functional pathways of amyotrophic lateral sclerosis signaling, Fc epsilon RI signaling, synaptogenesis signaling, fMLP signaling in neutrophils, RAC signaling, Reelin signaling in neurons, IL-8 signaling, endothelin-1 signaling, ephrin receptor signaling, and tumor microenvironment pathway. Exosome SGs in elastase induced AAA aortas had functional pathways, namely, CD28 signaling in T helper cells, interferon signaling, inducible nitric oxide synthase (iNOS) signaling, role of NFAT in regulation of immune response, regulation of IL-2 expression in activated and anergic T cells, Th1 pathway, systemic lupus erythematosus in B cell signaling, role of hypercytokinemia/hyperchemokinemia, T cell receptor signaling, and protein kinase C θ (PKCθ) signaling in T cells. WPB SGs in elastase induced AAA aortas had no significant functional pathways. Autophagy SGs in elastase induced AAA aortas had functional pathways, namely, neuroinflammation signaling, high mobility group box 1 (HMGB1) signaling, IL-17 signaling, systemic lupus erythematosus in B cell signaling, and cardiac hypertrophy signaling.

In addition, we determined whether elastase induced AAA shared SG pathways with that of Ang-II induced AAA. As shown in **Figure 5C**, elastase induced AAA shared six top canonical SG pathways with that of Ang-II induced AAA, namely, tumor microenvironment pathway, TREM1 signaling, role of hypercytokenemia/hyperchemokinemia in the pathogenesis of influenza, systemic lupus erythematosus in B cell signaling, dendritic cell maturation, and IL-17 signaling. Elastase induced AAA shared 0 top caspase 1 SGs with that of Ang-II induced AAA. Elastase induced AAA shared two top caspase 4 SG pathways with that of Ang-II induced AAA such as IL-8 signaling and RAC signaling. Elastase induced AAA shared one top exosome SG pathway with that of Ang-II induced AAA in role of hypercytokenemia/hyperchemokinemia in the pathogenesis of influenza. Elastase induced AAA shared five top autophagic SG pathways with that of Ang-II induced AAA, namely, cardiac hypertrophy, systemic lupus erythematosus in B cell signaling, neuroinflammation signaling, HMGB1 signaling, and IL-17 signaling.

Taken together, our results have demonstrated that *first*, elastase induced AAA aortas have more inflammatory and immune pathways than that of Ang-II induced AAA aortas. For example, elastase induced AAA aortas have IL-17, IL8, IL-6, T cell pathways and B cell pathways in various secretomes, which are well correlated with inflammation, influx of T cells to the aorta, upregulation of cytokines, and recruitment of leukocytes reported for elastase induced AAA mouse model (86); *second*, elastase induced AAA aortas have caspase 1 secretome and caspase 4 secretome with more T cell and B cell pathways, which are not found in atherosclerosis aortas, CKD aortas, and Ang-II induced AAA aortas; *third*, Elastase induced AAA shared 0 caspase 1 SG pathways with that of Ang-II induced AAA; and *fourth*, among the five secretomes examined, four secretomes such as canonical, caspase 1, caspase 4, exosome secretomes are significantly different in Ang-II induced AAA and elastase induced AAA.

### Most Disease-Upregulated Cytokines and Chemokines in Aorta Are Secreted *via* Canonical and Exosome Secretomes but not Caspase 1 and Caspase 4 Secretomes

Before caspase 1 secretome was reported in 2008 (61), it was been widely believed that most characterized cytokines and chemokines are secreted *via* canonical secretory pathway. However, it remains unknown that in mouse aortas affected by metabolic cardiovascular diseases whether well characterized cytokines and chemokines are all secreted *via* canonical secretory pathway. To examine this issue, we used the Ingenuity Pathway Analysis (IPA) designed list of 1,176 cytokines, chemokines and their interactors (https://www.proteinatlas.org/search/cytokine) as we reported (27). As shown in **Table 3**, in atherosclerotic aorta, 39 cytokines/interactors were upregulated in canonical secretome, no cytokines/interactors were upregulated in caspase 1 secretome, three cytokines/interactors were upregulated in caspase 4 secretome, 14 cytokines/interactors were upregulated in exosome secretome, one cytokine/interactor was upregulated in WBP, and three cytokines/interactors were upregulated in autophagy. In CKD aorta, 14 cytokines/interactors were upregulated in canonical secretome, no cytokines/interactors were upregulated in caspase 1 secretome, one cytokine/interactor was upregulated in caspase 4 secretome, 10 cytokines/interactors were upregulated in exosome secretome, no cytokines/interactors were upregulated in WBP, and two cytokines/interactors were upregulated in autophagy. In AAA, 10 cytokines/interactors were upregulated in canonical secretome, no cytokines/interactors were upregulated in caspase 1 secretome, no cytokines/interactors were upregulated in caspase 4 secretome, three cytokines/interactors were upregulated in exosome secretome, two cytokines/interactors were upregulated in WBP, and one cytokine/interactor was upregulated in autophagy.

**Table 3:** Most disease-upregulated cytokines from total 1176 genes in aortas are secreted via canonical and exosome secretory pathways but not caspase-1-, caspase-4-GSDMD secretory pathways.

	Canonical	Caspase 1	Caspase 4	Exosome	WPB	Autophagy
AS	39	0	3	14	1	3
MERS	25	5	6	52	0	1
AAA	10	0	0	3	2	1
CKD	14	0	1	10	0	2
Fistula	6	0	2	11	0	0

Numbers of upregulated cytokines in different disease conditions in each secretory pathway. Cytokine get from IPA analysis. P<0.05, LogFC>0.

Taken together, these results have demonstrated that 1) most IPA designated cytokines, chemokines and interactors are secreted *via* canonical (39/56 = 69.6%) and exosome (14/56 = 25%) SG pathways in atherosclerotic aorta; 2) most IPA designated cytokines, chemokines and interactors are secreted *via* canonical (14/25 = 56%) and exosome (10/25 = 40%) SG pathways in CKD aorta; 3) most IPA designated cytokines, chemokines and interactors are secreted *via* canonical (10/13 = 76.9%) and exosome (3/13 = 23%) SG pathways in AAA aorta; 4) no much cytokines, chemokines and interactors are secreted *via* caspase 1, and caspase 4 secretomes in those three aortic diseases; and 5) most of disease upregulated cytokines, chemokines and interactors in aorta are disease-specific.

### Canonical and Caspase 1 Secretome Play Roles at Early MERS-CoV (COVID19 Homologous Virus) Infection Whereas Caspase 4 and Exosome Secretome Play Roles in the Chronic Phase Infection; and the Early Upregulated Canonical and Caspase 1 Secretomes may Function as Drivers for Trained Immunity

**Figure 6:**
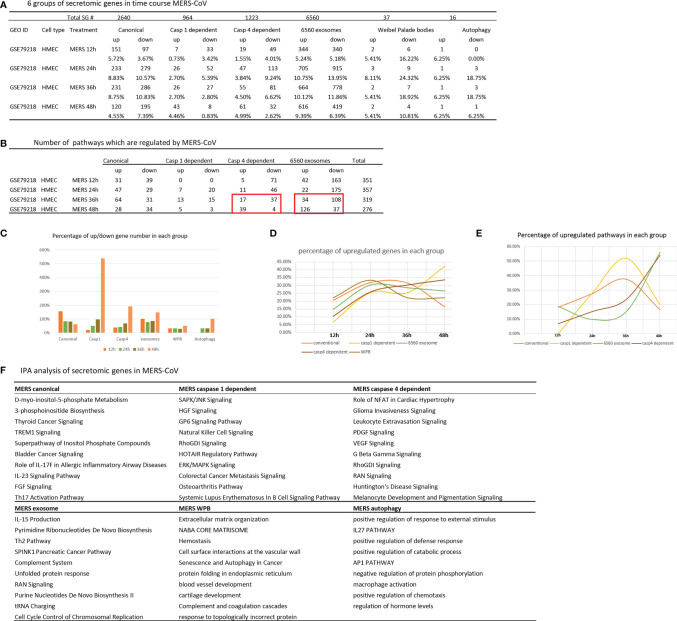
Canonical secretome and caspase 1 secretome play more roles at early MERS-CoV (COVID-19 homoglous virus) infection of human microvascular endothelial cells whereas caspase 4 secretome and exosome secretome play more roles in the late and chronic phase infection; and these results have suggested novel therapeutic targets. **(A)** Canonical secretion and exosomes are most important secretory pathways in MERS condition. Number of differentially modulated genes in each pathway. P <0.05, |LogFC|>1. **(B)** MERS 36 hour is a transition time point in this process. Number of pathways which are regulated in MERS condition. (|Z score|>1 ). **(C)** Percentage of up/down gene number in each group based on **(A)**. **(D)** Percentage of upregulated genes in each group. **(E)** Percentage of upregulated pathways in each group based on **(B)**. **(F)** IPA analysis of upregulated genes in each group. Top 10 pathways are shown. Z score>1.

Our previous report showed that large numbers of innate immune genes were modulated in human microvascular EC (HMECs) stimulated with wild-type MERS-CoV, a virus homologous to COVID-19 (35). However, canonical and noncanonical secretomic changes in HMECs infected with MERS-CoV remained unknown. As shown in **Figures 6A, B**, MERS-CoV infection of HMECs resulted in upregulation of 5.72, 8.83, 8.75, and 4.55% of canonical SGs at 12-, 24-, 36-, and 48-h post-infection, respectively. MERS-CoV infection of HMECs resulted in upregulation of 0.73, 2.7, 2.7, and 4.46% of caspase 1 SGs at 12-, 24-, 36-, and 48-h post-infection, respectively. MERS-CoV infection of HMECs resulted in upregulation of 1.55, 3.84, 4.5, and 4.99% of caspase 4 SGs at 12-, 24-, 36-, and 48-h post-infection, respectively. MERS-CoV infection of HMECs resulted in upregulation of 5.24, 10.75, 10.12, and 9.39% of exosome SGs at 12-, 24-, 36-, and 48-h post-infection, respectively. MERS-CoV infection of HMECs resulted in upregulation of 5.41, 8.11, 5.41, and 5.41% of WPB SGs at 12-, 24-, 36-, and 48-h post-infection, respectively. MERS-CoV infection of HMECs resulted in upregulation of 6.25, 6.25, 6.25, and 6.25% of autophagy SGs at 12-, 24-, 36-, and 48-h post-infection, respectively. In addition, as shown in **Figure 6C**, at 48 h post-infection, the SG modulation percentages were significantly increased in caspase 1, caspase 4, exosome, and autophagy secretomes but not canonical secretome, which were only increased in 12 h post-infection. Upregulated SG percentages in each group were similar in 12-, 24- and 36-h post-infection (**Figures 6D, E**). Canonical and caspase 1 SGs were upregulated at early time points but decreased at 36 h post-infection whereas exosome SGs and caspase 4 SGs were significantly increased starting from 36 h, suggesting that canonical and caspase 1 secretomes play more roles at early MERS-CoV infection of HMECs whereas caspase 4 and exosome secretomes play more roles in the late and chronic phase infection. These results suggested novel therapeutic targets.

The IPA analysis results of upregulated SGs in MERS-CoV-infected HMECs showed that the ten top pathways for each secretome were different, suggesting that during MERS-CoV infection of ECs, secretomes play their specific roles in secrete functional regulators (**Figure 6F**), which may serve as a potential mechanism underlying the pathogenesis of cytokine storm, EC dysfunction (28), immunothrombosis (88) and cardiovascular inflammation (89) in COVID-19 infection (35). Canonical SGs in MERS infection had different metabolic and inflammatory functional pathways, namely, D-myo-inositol 5-phosphate metabolism, 3-phosphoinsitide biosynthesis, thyroid cancer signaling, TREM1 signaling, super pathway of inositol phosphate compounds, bladder cancer signaling, roles of IL-17F in allergic inflammatory airway diseases, IL-23 signaling pathway, FGF signaling, and Th17 activation pathway. Canonical SGs in CKD had inflammatory functional pathways, namely, MIF regulation of innate immunity, TREM1 signaling, crosstalk between dendritic cells and natural killer cells, ILK signaling, neuroinflammation signaling, cardiac hypertrophy, hepatic fibrosis signaling, role of pattern recognition receptors, p38 MAPK signaling, and acute phase response signaling. Canonical SGs in AAA had less inflammatory but more neurosignaling, namely, agrin interactions at neuromuscular junction, ErbB signling, cholecystokinin/gastrin-mediated signaling, Her-2 signaling in breast cancer, amyotrophic lateral sclerosis signaling, antioxidant action of vitamin C, PPAR signalin, semaphoring neuronal repulsive signaling, PTEN signaling, and PPARa/RXRa activation.

In addition, we hypothesized that each disease uses several types of SGs for different functions. For example, as shown in **Figure 3A**, atherosclerosis had caspase 1 secretome for the functional pathways, namely, actin cytoskeleton signaling, leukocyte extravasation signaling, estrogen receptor signaling, actin nucleation by ARP–WASP complex, integrin signaling, synaptogenesis signaling, Rac signaling, Ephrin receptor signaling and colorectal cancer metastasis signaling. In addition, atherosclerosis had caspase 4 secretome for inflammation and B cell signaling, namely, roles of NFAT in regulation of immune response, B cell receptor, IL-8 signaling, neuroinflammation, role of PKR in interferon induction and antiviral response, Rac signaling, fMLP signaling, Ephrin receptor, endothelin-1 signaling, and systemic lupus erythematosus in B cell signaling.

Taken together, these results have demonstrated that 1) canonical and caspase 1 secretomes play more roles at early MERS-CoV infection of HMECs whereas caspase 4 and exosome secretomes play more roles in the late and chronic phase infection; 2) early secretomes, namely, canonical and caspase 1 secretomes may promote inflammation effectors (2), trained immunity (enhancing sustained/chronic inflammation and trained tolerance (inflammation resolution) (32, 65); 3) since ECs are one of the largest organs in the body (32), ECs are an immune organ in pathological conditions; and 4) SG upregulation in MERS-CoV infected HMECs may contribute significantly to cytokine storm, thrombosis and CVD related to COVID-19 infection (35).

### Venous ECs From Arteriovenous Fistula (AVF) Upregulates Genes in Five Secretomes, Resulting in Two Pathways in Canonical Secretome and 13 Pathways in Exosome Secretome; AVF Secretomes Have One Canonical and Five Exosome Pathways Unique; AVF Secretomes Share Pathways With That of Atherosclerosis Aorta, CKD Aorta, MERS-CoV Infected ECs, But Not With That of Ang-II Induced AAA Aorta

**Figure 7:**
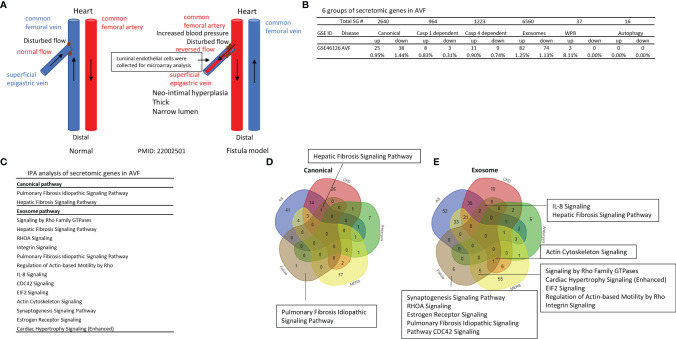
Venous ECs from arteriovenous fistula (AVF) upregulate SGs in five secretomes. **(A)** Experimental model of AVF with vascular anatomy (Red and Blue) and pathologic factors (Black). **(B)** Secretome analysis of venous endothelium during the formation of neointimal hyperplasia in a rat AVF model for kidney dialysis from GEO database (GSE46126) P <0.05 |LogFC|>1. **(C)** IPA analysis of each secretory pathway gene in AVF. Z score>1. **(D, E)** Venn diagram of upregulated canonical **(D)** and exosome **(E)** pathways in AVF.

Vascular shear stress results from flowing blood generated frictional force, which has major effects on vascular function. Complex blood flow patterns exert low or low oscillatory shear stress at branches and bends of arteries, this mechanical environment promotes vascular dysfunction and atherosclerosis (90). A significant rise of end-stage renal disease (21) in the world has resulted in increased placement of autogenous arteriovenous fistula (AVF) as the preferred type of vascular access for maintenance hemodialysis (91). Up to 60% of AVF fail to mature by 5 months, preventing their use for hemodialysis. The failed AVF defined by updated NKF-KDOQI guidelines (92) may result from shear stress in new branch and bend of artery-connected vein, changes of blood flow direction and arterial blood pressure actin on vascular wall (**Figure 7A**), inflammation, and inappropriate venous inward and outward remodelings (20), contributing to stenosis, access failure, significant patient burden and healthcare cost (93). RNA-Seq data identified that in AVF, gene expressions associated with inflammation, TGF-β; and cell death pathways are significantly modulated (94). Fractalkine receptor 1 (C-X3-C motif chemokine receptor 1, CX3CR1) blockade reduces venous neointimal hyperplasia (VNH)/venous stenosis (VS) formation in stenotic AVF samples by decreasing proinflammatory cues (95). However, inflammation related secretomes in AVF remain poorly characterized. We hypothesized that AVF injury significantly modulates secretomic gene expressions. To test this hypothesis, we examined the SG expression changes in venous ECs isolated during the formation of neointimal hyperplasia in a rat ATF model for kidney dialysis GEO database (GSE46126). As shown in **Figure 7B**, AVF upregulated 0.95% canonical secretome, 0.83% caspase 1 secretome, 0.90% caspase 4 secretome, 1.25% exosome secretome, 8.11% WPB secretome, and 0% autophagy secretome. The pathway analysis indicated that AVF canonical secretome had two functional pathways, namely, pulmonary fibrosis idiopathic signaling and hepatic fibrosis signaling; AVF exosome secretome had 13 functional pathways, namely, signaling by Rho family GTPases, hepatic fibrosis signaling, RHOA signaling, integrin signaling, pulmonary fibrosis idiopathic signaling, regulation of actin-based motility by Rho, IL-8 signaling, CDC42 signaling, EIF2 signaling, actin cytoskeleton signaling, synaptogenesis signaling, estrogen receptor signaling and cardiac hypertrophy signaling (**Figure 7C**). The Venn Diagram analysis showed that AVF canonical SGs had a unique pathway, pulmonary fibrosis idiopathic signaling; AVF canonical SGs had hepatic fibrosis signaling pathway shared with that of atherosclerotic aorta and CKD aorta; and AVF canonical SGs had no shared pathways with AAA aorta and MERS-CoV infected ECs (**Figure 7D**). In addition, AVF exosome SGs had five unique functional pathways such as synaptogenesis signaling, RHOA signaling, estrogen receptor signaling, pulmonary fibrosis idiopathic signaling, and CDC42 signaling; AVF exosome secretome had two pathways such as IL-8 signaling and hepatic fibrosis signaling shared with that of atherosclerosis aorta and CKD aorta; AVF exosome SGs had one pathway, actin cytoskeleton signaling shared with that of CKD aorta and MERS-CoV infected ECs; AVF exosome SGs had five pathways shared with MERS-CoV infected ECs, namely, Rho family GTPases, cardiac hypertrophy signaling, EIF2 signaling, regulation of actin-based motility by Rho, and integrin signaling (**Figure 7E**); and AVF exosome had no overlapped pathways with that of AAA aorta.

Taken together, our results have demonstrated that 1) AVF-venous ECs have significant modulations on the gene expressions of five secretomes but not the expression of autophagy SGs; 2) Upregulations of SGs in AVF-venous ECs result in two pathways in canonical SGs and 13 pathways in exosome SGs; 3) AVF-venous EC SGs have one canonical pathways and five unique exosome pathways; and 4) AVF-venous ECs SGs share two pathways with that of atherosclerotic aorta and CKD aorta; five pathways with MERS-CoV infected ECs, but share no any pathways with that of Ang-II induced AAA.

### Trained Immunity Participates in the Upregulation of Secretomes of Atherosclerotic Aorta, MERS-CoV Infection of ECs, Ang-II Induced AAA Aorta, and CKD aorta but Less in AVF Venous EC Reprogramming; Trained Immunity and Immune Metabolic Reprogramming Are in Different Paces in Various Diseases; NRF2 and NOX2 Regulate Trained Immunity Partially

**Figure 8:**
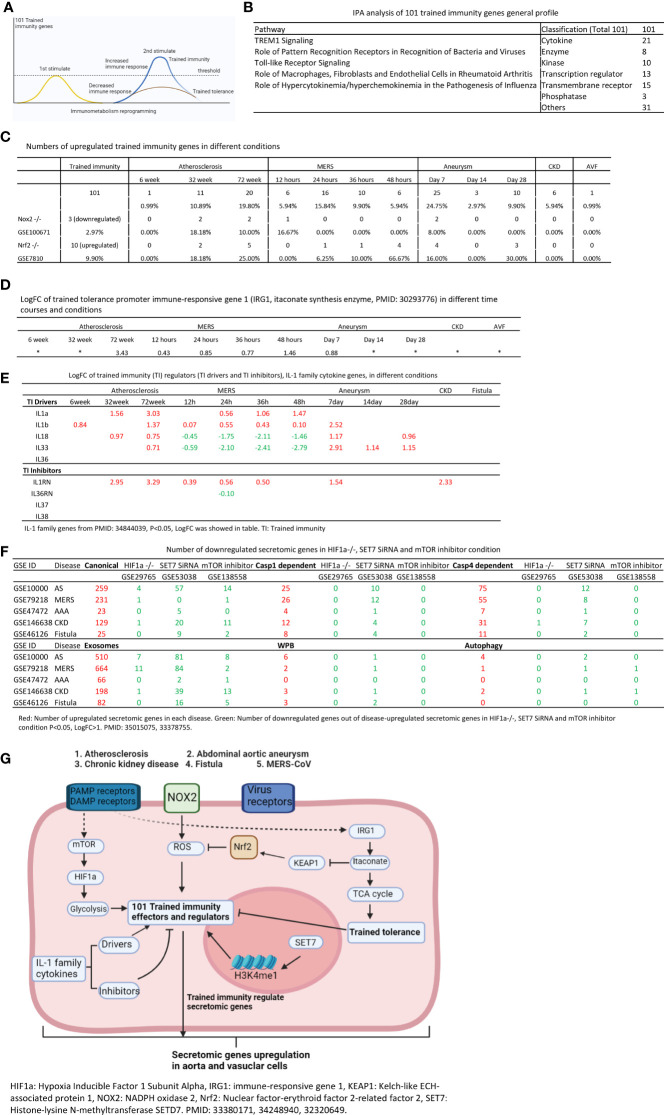
Increased trained immunity genes and decreased trained tolerance regulator immune-responsive gene 1 (IRG1) participate in upregulations of SGs in atherosclerotic, Ang-II induced AAA and CKD aortas, and MERS-CoV infected Ecs. **(A)** Model of trained immunity. **(B)** Trained immunity gene list from http://www.ieom-tm.com/tidb/browse. (After remove 17 duplicated gene by Excel, totally 101 genes involved) IPA analysis of 101 trained immunity genes. **(C)** Number of upregulated trained immunity gene in different time course disease and condition. P < 0.05, LogFC>1. **(D)** The LogFC of IRG1 in different time course and condition.(P < 0.05). **(E)** The expression of IL-1 family genes in different condition. IL-1 family genes from PMID: 34844039, P < 0.05, LogFC was showed in table. **(F)** Number of downregulated secretomic genes in HIF1a-/- (GSE29765), SET7 SiRNA (GSE53038) and mTOR inhibitor (GSE138558) condition. P < 0.05, LogFC>1. **(G)** Working model of trained immunity.

Secretomes and cytokines/chemokines play significant roles in promoting immune cell activation, differentiation (polarization), trans-differentiation (plasticity) in aorta and pathogenesis of various aortic pathologies (2, 8, 25, 50, 53). As we reported and reviewed, innate immune cells can develop exacerbated immunologic response and long-term inflammatory phenotype following brief exposure to endogenous or exogenous insults, which leads to an altered response towards a second challenge after the return to a nonactivated state. This phenomenon is known as trained immunity (TI) (32, 35, 52, 56, 59, 65, 96) (**Figure 8A**). We hypothesized that the trained immunity is one of driving mechanisms underlying the upregulation of SGs. To test this hypothesis, we examined the expression changes of 101 trained immunity genes (TIGs) from a comprehensive trained immunity database (http://www.ieom-tm.com/tidb/browse) in five vascular diseases. Of note, the IPA data showed that five pathways promoted by 101 TIGs, namely,TREM1 signaling, role of pattern recognition receptors in recognition of bacteria and viruses, TLR signaling, role of macrophage, fibroblasts and ECs in rheumatoid arthritis, and role of hypercytokinemia/hyperchemokinemia in pathogenesis of influenza. Furthermore, these TIGs further classified into cytokines (21 genes), enzymes (8 genes), kinases (10 genes), transcription factors (13 genes), transmembrane receptors (15 genes), phosphatases (3 genes), and others (31 genes) (**Figure 8B**). There were 1, 11, and 20 TIG upregulations in atherosclerosis at 6, 32, and 72 weeks, respectively. There were 6, 16, 10, and 6 TIG upregulations in MERS-CoV infected ECs at 12, 24, 36, and 48 h, respectively. There were 25, 3, and 10 TIG upregulations in Ang-II induced AAA aorta at days 7, 14, and 28, respectively. There were six TIG upregulation in CKD aorta, and one TIG upregulation, cardiovascular inflammation-promoting cytokine/adipokine (97) follistatin-like 1 (FSTL1) (98), in AVF venous ECs, respectively (**Figure 8C**).

The itaconate pathway and immune-responsive gene 1 (IRG1, itaconate synthesis enzyme) play significant role in promoting trained tolerance and inhibiting trained immunity and inflammation (99, 100) as shown in **Figure 8G**. We hypothesized that IRG1 expression is inhibited when the expressions of trained immunity genes are upregulated in most cases. As shown in **Figure 8D**, IRG1 was upregulated in 78 weeks atherosclerotic aorta, 48 h MERS-CoV infected ECs, and IRG1 expression was not significantly increased in MERS-CoV infected ECs at 12, 24, and 36 h post infection and day 7 AAA aorta. Of note, 24 to 36 h at MERS-CoV and day 7 AAA aorta, trained immunity gene upregulation were at the highest levels (**Figure 8C**), which supported our hypothesis in relatively acute inflammations in AAA aorta and MERS-CoV infection except in chronic atherosclerotic aorta. These data suggested a role of IRG1 in late atherosclerosis and late MERS-CoV infected endothelial cells.

We then asked whether ROS regulate the expression of trained immunity genes. To answer this question, we used two key ROS regulators, ROS generation enzyme nicotinamide adenine dinucleotide phosphate oxidase 2 (NOX2) deficient (knock-out, KO) datasets and ROS-suppressive transcription factor (TF) Nuclear factor erythroid 2-related factor 2 (NRF2, NFE2L2) KO datasets as model systems as we reported (21, 35). As shown in **Figure 8C**, there were three trained immunity genes downregulated in NOX2 KO datasets and 10 trained immunity gene upregulated in NRF2 KO dataset.

IL-1 family cytokines have been reported as drivers and inhibitors of trained immunity (101). Five IL-1 family cytokines promote trained immunity (TI) and four IL-1 family cytokines inhibit TI. We examined the expression changes of IL-1 family cytokines in different disease conditions. As shown in **Figure 8E**, four TI promoters were upregulated in atherosclerosis, three TI promoters were upregulated in AAA, and only two TI promoters were upregulated in MERS-CoV infected endothelial cells.

Recent progress reported that hypoxia inducible factor 1 alpha (HIF1α) (102), SET domain containing 7 histone lysine methyltransferase (SET7), and mechanistic target of rapamycin kinase (mTOR) promote trained immunity pathways (103) (**Figure 8G**). To determine whether HIF1α, SET7, and mTOR regulate the expression of upregulated secretomic genes in different disease conditions, we examined the disease-upregulated SG expression changes in HIF1α^−/−^ GEO database (GSE29765), SET7 SiRNA database (GSE53038), and mTOR inhibitor database (GSE138558). As shown in **Figure 8F**, in atherosclerosis, HIF1α^−/−^ downregulated 4 canonical SGs and 7 exosomes SGs. In MERS-CoV, HIF1α^−/−^ downregulated 1 canonical SG and 11 exosomes SGs. In AAA and AVF, HIF1a^−/−^ did not downregulate any SGs. HIF1a^−/−^ in CKD downregulated one canonical SG, one caspase 4 SG, and one exosome SG. However, SET7 siRNA in atherosclerosis downregulated 57 canonical SGs, 10 caspase 1 SGs, 12 caspase 4 SGs, 81 exosomes SGs, one WBP SG, and two autophagy SGs. SET7 siRNA in MERS-CoV infection downregulated 12 caspase 1 SGs, 8 caspase 4 SGs, 84 exosomes SGs, one WBP SG, and one autophagy SG. In AAA, SET7 siRNA downregulated 5 canonical SGs, one caspase 1 SG, one caspase 4 SG, and two exosomes SGs. In CKD, SET7 siRNA downregulated 20 canonical SGs, 4 caspase 1 SGs, 7 caspase 4 SGs, 39 exosomes SGs, one WBP SG, and one autophagy SGs. In addition, SET7 siRNA in AVF downregulated 9 canonical SGs, 4 caspase 1 SGs, two caspase 4 SGs, 16 exosomes SGs, and two WBP SGs. Furthermore, mTOR inhibitor downregulated 14 canonical SGs and 8 exosomes SGs in atherosclerosis, but in MERS infection, mTOR downregulated one canonical SG, two exosomes SGs, and one autophagy SG. In AAA, mTOR inhibition downregulated only one exosomes SG. However, mTOR inhibition in CKD downregulated 11 canonical SGs, 13 exosomes SGs, and one autophagy SG. Finally, mTOR inhibition in AVF downregulated two canonical SGs and 5 exosomes SGs. These results indicated that SET7 siRNA downregulated more canonical, caspase 1, caspase 4, and exosomes SGs than HIF1a^−/−^ and mTOR inhibition.

Taken together, these results have demonstrated that *first*, trained immunity participates in upregulation of secretomes in atherosclerotic aorta, MERS-CoV infection of ECs, Ang-II induced AAA, and CKD aorta but less in AVF venous EC reprogramming; *second*, trained immunity gene upregulation peak time courses are different: atherosclerosis at 72 weeks, MERS-CoV infection of ECs at 24 h, Ang-II induced AAA at day 7 reach their peak time in upregulating trained immunity genes, suggesting that trained immunity establishment and immune metabolic reprogramming in aorta and aortic ECs are in different pace in various diseases; *third*, NOX2 upregulates 3 out of 101 trained immunity and NRF2 suppresses 10 out of 101 trained immunity genes, suggesting that ROS partially regulate trained immunity; and *fourth*, SET7 deficiency play downregulated more canonical, caspase 1, caspase 4, and exosome SGs than HIF1a^−/−^ and mTOR inhibition in atherosclerosis, CKD, AAA, MERS-CoV infection, and AVF.

### ROS-Suppressing NRF2 Plays More Significant Roles in Downregulating SGs Than That of ROS Generating NOX2 in Upregulating SGs in Aortic and Vascular Pathologies; NRF2 Plays More Roles in Downregulating SGs in Canonical, Caspase 4 and Exosome Secretomes Than That in Caspase 1 Secretome; and NOX2 Plays More Roles in Upregulating SGs in Canonical and Exosome Secretomes Than That in Caspase 1 and Caspase 4 Secretomes

We hypothesized that ROS regulate the expression of secretomes in aortic pathologies, EC infection by MERS-CoV and AVF venous EC reprogramming. To examine this hypothesis, we first examined the upregulation of 165 ROS regulators in pathologies. Various aortic and vascular cell pathologies resulted in upregulation of ROS regulators: 33 ROS regulators were upregulated in atherosclerosis, 11 ROS regulators were upregulated in CKD-aorta, 15 ROS regulators were upregulated in MERS-CoV infected ECs, but no ROS regulators were upregulated in fistula venous EC reprogramming (**Table 4**). To determine the causative effects of ROS regulators in contributing to the upregulation of SGs in aortic and vascular cell pathologies, we used two key ROS regulators, NOX2 deficient datasets and NRF2 KO datasets as model systems as we reported (21, 35). As shown in **Table 2**, among 259 canonical SGs upregulated in atherosclerotic aorta, 5 SGs (1.93%, LogFC <−1) were downregulated in NOX2^−/−^ dataset; and 47 SGs (18.15%, LogFC >1) were upregulated in NRF2^−/−^ dataset. Among 129 canonical SGs upregulated in CKD aorta, 4 SGs (3.10%, LogFC <−1) were downregulated in NOX2^−/−^ dataset; and 13 SGs (10.08%, LogFC >1) were upregulated in NRF2^−/−^ dataset. Among 23 canonical SGs upregulated in AAA aorta, 0 genes (0%, LogFC <−1) were downregulated in NOX2^−/−^ dataset; and 1 SG (4.35%, LogFC >1) were upregulated in NRF2^−/−^ dataset. Among 120 canonical SGs upregulated in MERS-CoV infected ECs, two SGs (1.67%, LogFC <−1) were downregulated in NOX2^−/−^ dataset; and 11 SGs (9.17%, LogFC >1) were upregulated in NRF2^−/−^ dataset. Among 25 canonical SGs upregulated in AVF venous ECs, only one gene (4%, LogFC <−1) was downregulated in NOX2^−/−^ dataset; and two SGs (8%, LogFC >1) were upregulated in NRF2^−/−^ dataset.

**Table 4:** Upregulated secretomic genes in each secretory pathway were screened in Nox2-/- (GSE100671) or Nrf2-/- (GSE7810) dataset.

ID	Disease	ROS regulator (165)	LogFC<-1	-1<LogFC<0	LogFC>1	1>LogFC>0		LogFC<-1	-1<LogFC<0	LogFC>1	>1LogFC>0
			Canonical	Nox2 -/-	Nox2 -/-	Nrf2 -/-	Nrf2 -/-	Caspase 1	Nox2 -/-	Nox2 -/-	Nrf2 -/-	Nrf2 -/-
**GSE10000**	Atherosclerosis	33	259	5	42	47	59	25	0	7	2	10
		20.00%		1.93%	16.22%	18.15%	22.78%		0.00%	28.00%	8.00%	40.00%
**GSE146638**	CKD	11	129	4	15	13	22	12	0	4	1	2
		6.67%		3.10%	11.63%	10.08%	17.05%		0.00%	33.33%	8.33%	16.67%
**GSE47472**	Aneurysm	0	23	0	3	1	2	4	0	1	2	0
		0.00%		0.00%	13.04%	4.35%	8.70%		0.00%	25.00%	50.00%	0.00%
**GSE79218**	MERS	15	120	2	10	11	17	43	0	6	3	3
		9.09%		1.67%	8.33%	9.17%	14.17%		0.00%	13.95%	6.98%	6.98%
**GSE46126**	Fistula	0	25	1	1	2	6	8	0	1	0	3
		0.00%		4.00%	4.00%	8.00%	24.00%		0.00%	12.50%	0.00%	37.50%
				LogFC<-1	LogFC<0	LogFC>1	LogFC>0		LogFC<-1	LogFC<0	LogFC>1	LogFC>0
**ID**	Disease		Caspase4	Nox2 -/-	Nox2 -/-	Nrf2 -/-	Nrf2 -/-	Exosome	Nox2 -/-	Nox2 -/-	Nrf2 -/-	Nrf2 -/-
**GSE10000**	Atherosclerosis		75	3	26	17	29	510	13	114	86	117
				4.00%	34.67%	22.67%	38.67%		2.55%	22.35%	16.86%	22.94%
**GSE146638**	CKD		31	0	8	6	6	198	7	32	31	39
				0.00%	25.81%	19.35%	19.35%		3.54%	16.16%	15.66%	19.70%
**GSE47472**	Aneurysm		7	0	0	2	1	66	0	10	7	3
				0.00%	0.00%	28.57%	14.29%		0.00%	15.15%	10.61%	4.55%
**GSE79218**	MERS		61	0	10	6	3	616	11	104	37	37
				0.00%	16.39%	9.84%	4.92%		1.79%	16.88%	6.01%	6.01%
**GSE46126**	Fistula		11	0	2	2	2	82	2	13	2	14
				0.00%	18.18%	18.18%	18.18%		2.44%	15.85%	2.44%	17.07%

P < 0.05. PMID: 31153039. These results have demonstrated that NOX2 may promote trained immunity via increasing reactive oxygen species (ROS) and promote disease-upregulated secretomic genes; and NRF2 as anti-oxidant transcription factor may suppress ROS and inhibit trained immunity and downregulate disease-upregulated secretomic genes.

Among caspase 1 SGs upregulated in atherosclerotic aorta (25 genes), CKD aorta (12 genes), AAA aorta (4 genes), MERS-CoV infected ECs (43 genes) and AVF venous EC reprogramming (8 genes), 0 genes were downregulated (LogFC <−1) in NOX2^−/−^ dataset; and 8% (atherosclerotic aorta), 8.33% (CKD aorta), 50% (AAA aorta), 6.98% (MERS-CoV ECs) and 0% genes (AVF venous ECs) (LogFC >1) were upregulated in NRF2^−/−^ dataset, respectively. Among caspase 4 SGs upregulated in atherosclerotic aorta (75 genes), CKD aorta (31 genes), AAA aorta (7 genes), MERS-CoV infected ECs (61 genes) and AVF venous EC reprogramming (11 genes), 4% genes in atherosclerotic aorta were downregulated but no SGs were downregulated in other datasets (LogFC <−1) in NOX2^−/−^ dataset; and 22.67% (atherosclerotic), 19.35% (CKD), 28.57% (AAA), 9.84% (MERS-CoV ECs) and 18.18% SGs (AVF venous ECs) (LogFC >1) were upregulated in NRF2^−/−^ dataset, respectively. Among exosome SG upregulated in atherosclerotic aorta (510 SGs), CKD aorta (198 SGs), AAA aorta (66 SGs), MERS-CoV infected ECs (616 SGs) and AVF venous EC reprogramming (82 SGs), 2.55% SGs in atherosclerotic aorta, 3.54% SGs in CKD aorta, 0% SGs in AAA aorta, 1.79% SGs in MERS-CoV ECs, and 2.44% SGs in AVF venous ECs were downregulated (LogFC <−1) in NOX2^−/−^ dataset; and 16.86% (atherosclerotic), 15.66% (CKD), 10.61% (AAA), 6.01% (MERS-CoV ECs) and 2.44% SGs (AVF venous ECs) (LogFC >1) were upregulated in NRF2^−/−^ dataset, respectively.

Taken together, these results have demonstrated that: 1) NRF2 plays more significant roles in downregulating SGs than that of NOX2 in upregulating SGs in aortic and vascular pathologies; 2) NRF2 plays more roles in downregulating SGs in canonical, caspase 4 and exosome secretomes than that in caspase 1 secretome; and 3) NOX2 plays more roles in upregulating SGs in canonical and exosome secretomes than that in caspase 1 and caspase 4 secretomes.

## Discussion

Our previous work and those of others have demonstrated that ECs are innate immune cells in pathological conditions stimulated by DAMPs/PAMPs. Significant progress has been made to demonstrate that vascular cells and recruited immune cells in aortas in response to DAMPs and PAMPs undergo differentiation and trans-differentiation to promote the pathogenesis of atherosclerosis, CKD-enhanced aortic inflammation, AAA and AVF reprogramming. However, a key question remains to be addressed, by what stimuli, in addition to DAMPs/PAMPs, do those vascular cells and recruited immune cells differentiate and trans-differentiate. To answer the question, we performed extensive transcriptomic analyses of six secretomic gene lists in many microarray datasets deposited in the NIH/NCBI GeoDatasets database and the EMBL-EBI-ArrayExpress repository, and made the following findings: 1) 53.7% out of 21,306 human protein-encoding genes are classified into six secretomes; among which four secretomes, canonical, caspase 1, caspase 4 and exosomes, are the large secretomes with >900 proteins; and six secretomes are ranked from the highest specificity to the lowest as exosome (83%) > canonical (68%) > caspase 1 (56%) > caspase 4 (49%) > autophagy (37.5%) > WPB (8%); 2) Atherosclerosis, CKD, and AAA significantly modulate six secretomes in aortas; and MERS-CoV infection in human ECs and Angiotensin-II treatment in VSMCs also modulate six secretomes; 3) Atherosclerotic aortas upregulate T cell and B cell adaptive immune secretomic genes (SGs); CKD aortas upregulate SGs for cardiac hypertrophy, hepatic fibrosis and senescence; and AAA aortas upregulate SGs for neuromuscular signaling, protein catabolic process and Fcγ receptor-mediated phagocytosis; 4) Canonical secretome in atherosclerotic aortas upregulate pattern recognition receptors, leukocyte extravasation at 6 weeks, T cell exhaustion, fibrosis and neuroinflammation at 32 and 78 weeks; caspase 1, and caspase 4, GSDMD secretomes have no functions at 6 weeks, but have functions of cancer metastasis, NFAT and B cell signaling at 32 and 78 weeks, respectively; and exosome secretome has eNOS, and calcium signaling at 6 weeks but GF-CSF, NFAT, and T cell signaling at 32 and 78 weeks; 5) In Ang-II induced AAA, canonical, caspase 4-GSDMD, and exosome secretomes have two peaks (three-phases) of high (day 7)-low (day 14)-high (day 28) expression patterns; and caspase 1-GSDMD, Weibel–Palade body, and autophagy secretomes are only functional at the day 7; 6) Elastase-induced AAA aortas have more inflammatory and immune pathways than that of Ang-II induced AAA aortas; 7) Most disease-upregulated cytokines and chemokines in aorta are secreted *via* canonical and exosome secretory pathways but not caspase 1 and caspase 4-GSDMD secretory pathways; 8) Canonical and caspase 1 secretomes play roles at early MERS-CoV infection whereas caspase 4 and exosome secretomes play roles in the chronic phase infection; and the early upregulated canonical and caspase 1 secretomes may function as drivers for trained immunity; 9) Venous ECs from AVF upregulates genes in five secretomes, resulting in two pathways in canonical secretome and 13 pathways in exosome secretome; 10) Trained immunity participates in the upregulation of secretomes of atherosclerotic aorta, MERS-CoV infection of ECs, Ang-II induced AAA aorta, and CKD aorta but less in AVF venous EC reprogramming; trained immunity and immune metabolic reprogramming are in different pace in various diseases; NRF2 and NOX2 partially regulate trained immunity; and 11) ROS-suppressing transcription factor NRF2 plays more significant roles in downregulating secretomic genes than that of ROS generating enzyme NOX2 in upregulating secretomic genes in aortic and vascular pathologies; NRF2 plays more roles in downregulating genes in canonical, caspase 4 and exosome secretomes than that in caspase 1 secretome; and NOX2 plays more roles in upregulating genes in canonical and exosome secretomes than that in caspase 1 and caspase 4 secretomes.

**Figure 9:**
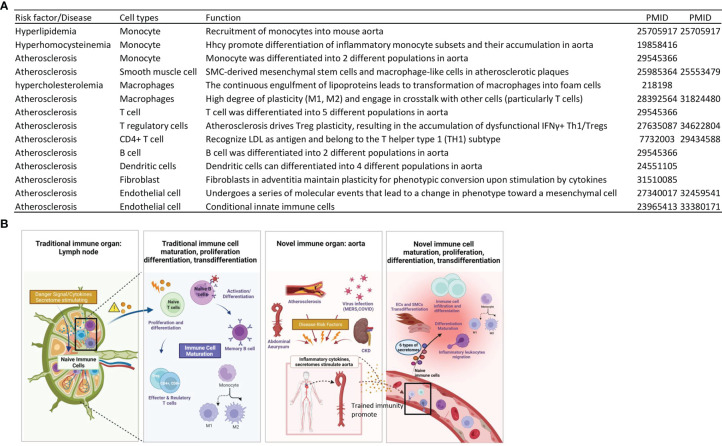
Under pathological conditions, immune cells in aortas undergo maturation, activation, phenotypic switch and trans-differentiation, which justifies that aorta serves as an immune organ. **(A)** Literature based findings indicate that aorta may play a role as immune organ. **(B)** A new working model (created in BioRender.com) has indicated that similar to lymph nodes, a prototypic immune organ, which provide a niche for immune cell maturation, differentiation and activation, aortas sin pathologies serve as a novel immune organ for immune cells and vascular cells to get activated, matured, differentiated and trans-differentiated.

The major rationale for classifying lymph nodes and spleen as peripheral immune organs is that these organs provide a niche for immune cell maturation, differentiation, and activation (104). If we hold the same criteria and examine recent progresses in the aorta field, we will have no difficulties to conclude that aorta is an immune organ in pathologies. Previously, we reported that ECs including human aortic ECs are innate immune cells (3, 32, 35). Our RNA-Seq paper showed that human aortic ECs activated by LysoPC are trans-differentiated into innate immune cells by upregulating EC adhesion molecules, cytokines/chemokines, additional DAMP receptors, MHC molecules (51); co-stimulation receptors and immune checkpoints (57) are upregulated in other ECs stimulated by proinflammatory cytokines such as TNF-α and IFN-γ (57); and a comprehensive list of innate immune regulators (innatome) are significantly upregulated in 28 various types of ECs stimulated by conditional DAMPs and PAMPs including SARS-CoV-2 (COVID-19) (35). Others reported that during atherogenesis, VSMCs undergo phenotypic switching into macrophage-like inflammatory cells (105). We also reported that VSMCs undergo phenotypic switch stimulated by uremic toxin serum from patients with CKD (17). Moreover, CD4^+^Foxp3^+^ Tregs in aorta experience T helper cell (Th) lineage plasticity into Th1-Tregs, Th2-Tregs, Th-17-Tregs, and Tfh-Tregs (106), and antigen presenting cell (APC)-Tregs (22). We reported that Treg plasticity in mouse aorta is suppressed by immunosuppressive cytokine IL-35 (25, 53, 55, 107). Studies showed that in both human and experimental AAAs, prominent inflammatory cell infiltration, such as CD4^+^ T cells and macrophages, undergo phenotypic modulation based on microenvironmental cues. The skew to proinflammatory phenotypes alters disease progression and plays a role in causing chronic inflammation (108). Atherosclerosis immune responses are controlled by artery tertiary lymphoid organs (ATLOs) in the adventitial connective tissue adjoining arteries *via* VSMC lymphotoxin b Receptors (109, 110). ATLOs recruit large numbers of B-1 cells whose subtypes are skewed toward IL-10^+^ B-1b cells versus IL-10^-^ B-1a cells (110). Germinal center-derived IgG antibodies promote the size and stability of atherosclerosis plaques, through promoting arterial SMC proliferation and maintaining the molecular identity of the aorta (111). Single cell RNA sequencing identified 17 types of aortic cells including 11 principal leukocyte clusters (112), which include five types of macrophages (mac) such as interferon-inducible (IFNIC)-mac, inflammatory mac, monocyte (mono)/mac/dendritic cell (DC)/CD209a (monocyte-derived DC) (113), resident mac, triggering receptor expressed on myeloid cells-2 (Trem2) foamy mac. These findings suggest immune cell activation, polarization, differentiation/trans-differentiation and diversity in aorta (114). Taken together, all these findings suggest that under pathological conditions, immune cells in aortas undergo maturation, activation, phenotypic switch and trans-differentiation, which justifies that an aorta serves as an immune organ (**Figure 9A**).

The original microarray experiments deposited in the NCBI database used mouse aortas in different pathologies, which prevented us from comparing the secretomic gene expressions in the same -omics analysis settings. In addition, since the transcriptomic experiments were performed in many different laboratories, variations between the results cannot be ruled out due to different experimental conditions. Of note, a similar method was used in **Figure 4**b of the Nature Immunology paper from Drs. Mathis and Benoist teams (115). Although our database mining approach was not ideal, however, as the first step to fill in the important knowledge gap this approach was justified, which becomes a common practice that we (21) and others (116) often used. Another limitation of the current study is that due to the low throughput nature of current verification techniques in the laboratories, we could not verify the results we identified with the analyses of high throughput data. Of note, many of those high throughput datasets have been published in papers of other investigators with experimental verification on some data. We acknowledge that carefully designed *in vitro* and *in vivo* experimental models will be needed to continuously verify the findings presented here.

To summarize our findings here, we propose a new working model (**Figure 9B**). *First*, peripheral lymphoid organ, lymph nodes, where naïve lymphocytes and other immune cells become activated, or differentiated/polarized into effector subsets. By comparison, in aorta during atherosclerotic and other pathologies, endothelial cells get activated, vascular smooth muscle cells (VSMCs) become phenotypical switched into macrophages/monocytes-like VSMCs, monocytes become Ly6C^+/high^ proinflammatory ones, macrophages are polarized into M1 and other effector subsets, CD4^+^ Tregs become plastic into Th1-like, Th17-like and other status in addition to maintain their immunosuppressive functions; *second*, significantly upregulated six types of secretomes, namely, canonical, caspase 1-GSDMD, caspase 4-GSDMD, exosome, WPB, and authophagy, promote the establishment of niches for immune activation, differentiation (polarization), and trans-differentiation (plastic); and *third*, trained immunity promoters, IL-1 family cytokine trained immunity promoters, HIF1a, SET7, mTOR, and ROS generator NOX2 (60) as underlying mechanisms contribute to the upregulation of aortic and vascular cell secretomic genes whereas trained tolerance promoter IRG1, IL-1 family cytokine TI inhibitors and anti-oxidant transcription factor NRF2 are underlying mechanisms for inhibition of secretomic gene upregulation in pathologies. Recently several single cell RNA-Seq analyses (117) deposited in the Single Cell Porter database (https://singlecell.broadinstitute.org/single_cell?type=study&page=1&terms=aorta) in identifying numerous subtypes of monocytes, endothelial cells, fibroblasts support this new working model. Therefore, aortas can be classified into an immune organ, which express immune regulatomic genes such as receptors for virus infections, DAMPs/PAMPs (PPRs), cytokines (54), chemokines (50), cell death (8), growth factors (50), immune checkpoints (57), and regulators for trained immunity (59)-related metabolic and epigenetic reprogramming (118), and play significant roles in vascular homeostasis, inflammatory cell recruitment, myelopoiesis, innate immunity, cell death, thromboembolism, cytokine storms and cardiovascular comorbidities of COVID-19 and other viral infections. We believe that extensive future work are needed to re-examine the high-throughput results reported here with relatively low throughput methods currently in most laboratories. Nevertheless, our findings provide novel insights on the roles of upregulated secretomic genes in the pathogenesis of various aortic and vascular inflammatory diseases and also new targets for the future therapeutic interventions for inflammations, CVDs, autoimmune diseases, transplantation and cancers.

## Data Availability Statement

The original contributions presented in the study are included in the article/supplementary material. Further inquiries can be directed to the corresponding author.

## Author Contributions

YL carried out data collections, data analyses and drafted the manuscript. YuS, KX, FS, YiS, CDIV, SW, WH, JY, SPK, JRB, RIVP, JS, XJ, and HW provided material input. XY supervised experimental design and data analyses. XY edited the manuscript. All authors listed have made a substantial, direct, and intellectual contribution to the work and approved it for publication.

## Funding

This work was supported by the National Institutes of Health Grants to XY.

## Conflict of Interest

The authors declare that the research was conducted in the absence of any commercial or financial relationships that could be construed as a potential conflict of interest.

## Publisher’s Note

All claims expressed in this article are solely those of the authors and do not necessarily represent those of their affiliated organizations, or those of the publisher, the editors and the reviewers. Any product that may be evaluated in this article, or claim that may be made by its manufacturer, is not guaranteed or endorsed by the publisher.
